# Tensile Behavior of Pre-Embedded Titanium-Alloy Joints in Filament-Wound Carbon-Fiber Composite Tubes After Low-Temperature Conditioning

**DOI:** 10.3390/ma19214526

**Published:** 2026-10-27

**Authors:** Xiaoyong Liu, Zifei Ma, Siyuan Li, Cheng Guo, Haibo Luo, Jun Wu

**Affiliations:** 1 Aerospace Information Research Institute, Chinese Academy of Sciences, Beijing 100094, China; liuxiaoyong23@mails.ucas.ac.cn (X.L.); mazifei22@mails.ucas.ac.cn (Z.M.); lisiyuan242@mails.ucas.ac.cn (S.L.); guocheng25@mails.ucas.ac.cn (C.G.); luohb@aircas.ac.cn (H.L.); 2 School of Space Exploration, University of Chinese Academy of Sciences, Beijing 100040, China

**Keywords:** stratospheric airship, filament-wound carbon-fiber composite tube, pre-embedded titanium-alloy joint, low-temperature conditioning, axial tension, pull-out failure

## Abstract

Pre-embedded metal inserts provide a direct load-introduction path for filament-wound composite tubes without cutting the load-carrying fibers. This study evaluates the room-temperature axial tensile response of T700 carbon-fiber/TDE86 epoxy tubes containing grooved, internally threaded TC4 titanium-alloy inserts after one programmed low-temperature conditioning cycle and separately examines a small ambient end-enlarged configuration. Twenty-one destructive test records were analyzed: nine ambient constant-section (“equal-section”) specimens, nine independent equal-section specimens after a 25 → −70 → 25 °C chamber-setpoint cycle followed by 24 h at room temperature, and three ambient end-enlarged specimens. At 1 mm/min, the three cohorts had mean peak loads of 81.39, 81.31, and 88.26 kN and coefficients of variation of 14.04%, 8.70%, and 7.85%, respectively. The −0.07 kN difference between the two independent equal-section cohort means does not establish equivalence or absence of a conditioning effect, and the three-specimen end-enlarged comparison does not isolate a causal geometry effect. Macroscopic failure was dominated by insert pull-out accompanied by visible adhesive/interfacial separation, laminate-layer damage, attached fragments, and local splitting. Because local slip, bond-line deformation, and system compliance were not measured, equilibrium and competing limit states are used only as a qualitative framework. The results concern post-conditioning room-temperature response, not in situ performance at −70 °C.

## 1. Introduction

Long-endurance stratospheric airships require lightweight load-bearing frames, but the efficiency of filament-wound carbon-fiber-reinforced polymer (CFRP) tubes depends on how concentrated loads enter the continuous-fiber shell. Drilled and bolted joints provide a direct mechanical path but interrupt fibers and can promote bearing damage, splitting, and delamination; their response depends strongly on the fastener and laminate architecture [1,2,3,4,5,6]. Adhesive and hybrid joints preserve more of the fiber path, yet their strength and fracture mode depend on the overlap geometry, adherend stiffness, bond-line construction, surface preparation, and environment [7,8,9,10,11,12,13,14,15,16,17,18]. Neither family by itself resolves the combined requirement of uninterrupted winding and a mechanically robust end connection. Studies on Ti/CFRP hybrid inserts and adhesively bonded FRP tubular socket joints further show that the joint architecture and load-transfer design are central to the performance of composite-to-metal and composite-tube connections [19,20].

Tube studies further show that the winding angle, mosaic pattern, tension, compaction, ply count, geometry, loading mode, and environmental history affect the strength, damage, and scatter [21,22,23,24,25,26,27,28,29,30,31,32]. For an embedded end joint, these variables are not background details: they act in the same small load-transfer region in which the adhesive, grooves, and wound plies interact. Consequently, differences between small independently manufactured cohorts can be confounded by manufacturing variation, and an unmatched comparison supports description and hypothesis generation rather than attribution to one environmental or geometric factor.

The closest architectural precedent is Luo et al. [33], who introduced a grooved TC4 titanium-alloy insert integrated into a T700 carbon-fiber/TDE86 epoxy tube during wet filament winding. Their D31 room-temperature experiments and finite-element analysis established the feasibility of the baseline connection and proposed a progression from adhesive-region damage to ±30°-ply damage and interlaminar delamination. However, that study did not analyze the D33/D35 ambient records, a post-conditioned cohort, or a locally end-enlarged configuration. In the present data set, D31-RT-A to D31-RT-C are therefore disclosed as reanalyzed baseline records from Ref. [33], whereas the other 18 records were not analyzed in that paper.

The closest environmental comparators answer related but non-identical questions. Kaiser et al. [34] tested conventional CFRP/titanium tubular adhesive lap joints at cold, room, and elevated temperatures, thereby demonstrating temperature-dependent competition among adhesive, laminate, and metal failure modes. Luo et al. [35] studied adhesively bonded T700/C204 composite and TC4 tubes under room-temperature, in situ −65 °C, and room–low–room-temperature histories; their joints used a different lap-joint architecture, resin system, J-250 film-bond construction, cure/pressure schedule, and thermal protocol. Those studies establish that the temperature path, test temperature, and joint architecture matter, but they do not quantify the post-conditioning response of the present grooved co-wound insert. Coupon and double-lap studies [36,37,38,39,40,41,42] provide further mechanism-level evidence for environmental sensitivity but cannot substitute for component-level evidence. Table S1 provides a structured comparison of the joint architecture, material system, thermal history, test temperature, evidence type, and remaining limitations.

This distinction defines the unresolved question more precisely: after one 25 → −70 → 25 °C chamber-setpoint cycle, removal from the chamber, and 24 h at room temperature, what axial peak-load distribution and macroscopic failure family are observed for an independently manufactured cohort of this specific joint family? A secondary explicitly descriptive question is what response is observed for three ambient end-enlarged specimens. The available design cannot establish in situ strength at −70 °C, statistical equivalence to ambient controls, repeated-cycle durability, or an isolated causal effect of end enlargement.

Accordingly, this study reports the peak load, nominal axial stress in the free tube section, load–machine-displacement history, and specimen-level macroscopic failure observations for nine ambient equal-section specimens, nine post-conditioned equal-section specimens, and three ambient end-enlarged specimens. Here, “equal-section” denotes a constant nominal tube section along the shell/gauge region; it does not mean that the tube and insert have identical cross sections. Analyses of tubular bonded joints and progressive damage [43,44,45,46,47,48,49,50] are used only to organize a qualitative interpretation of the mixed pull-out morphology because the local slip, bond-line deformation, groove-fill quality, and load-train compliance were not measured [51,52]. The contribution is therefore a component-level data set with clearly stated evidential limits, explicit separation from Ref. [33], and a transparent comparison framework—not a new quantitative constitutive or causal model.

## 2. Materials and Methods

### 2.1. Joint Architecture and Specimen Series

**Figure 1:**
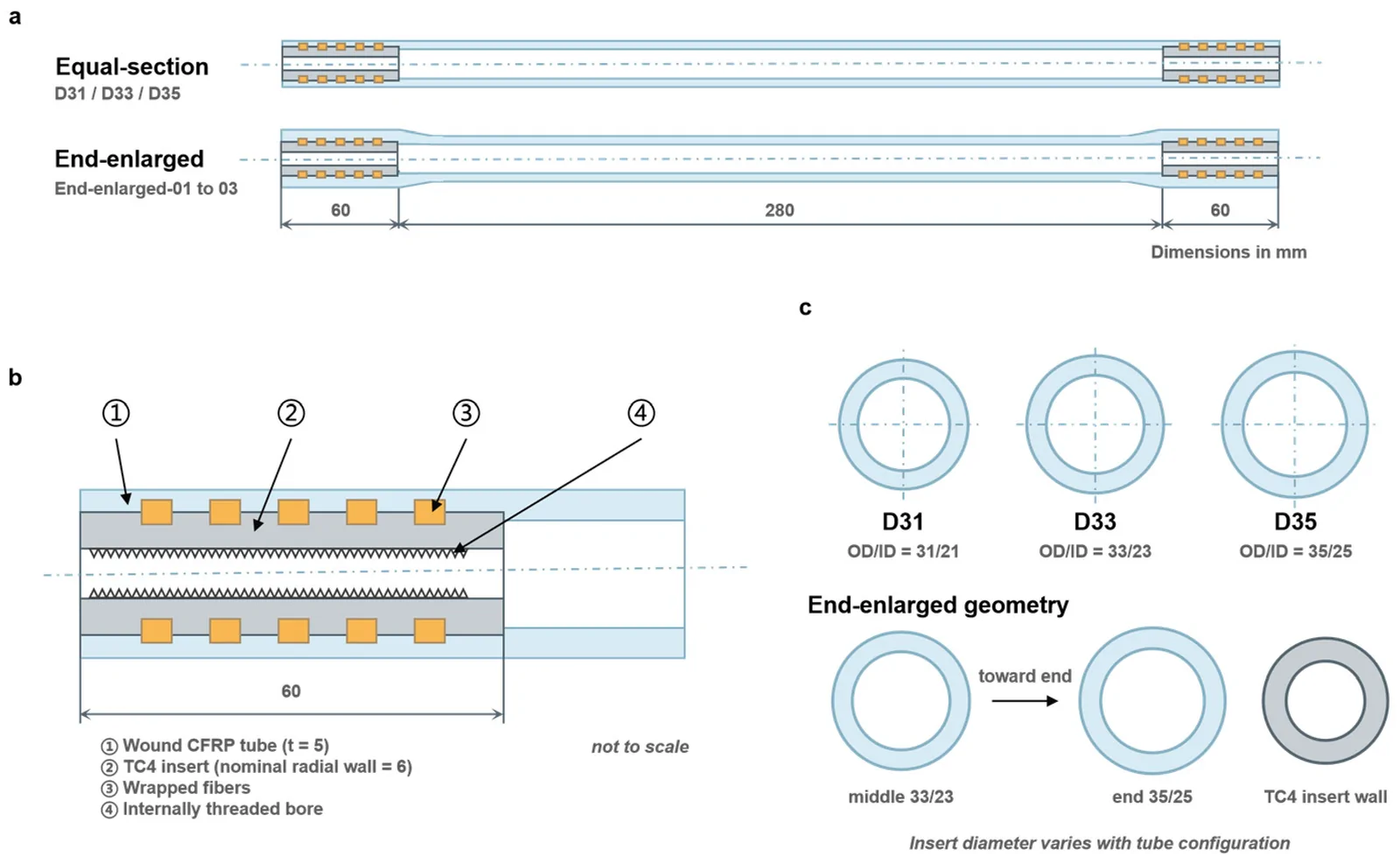
Specimen geometry and dimensional definitions: (**a**) axial layouts of the equal-section and end-enlarged specimens; (**b**) schematic cutaway of the pre-embedded grooved TC4 insert; and (**c**) nominal composite-tube sections. The tube and non-threaded insert dimensions are configuration-specific and are tabulated in Table S2a; dimensions are in millimeters, and the schematic is not to scale.

The joint configuration followed Luo et al. [33]. TC4 denotes the titanium-alloy grade used for the inserts. A grooved internally threaded TC4 insert supplied by Changlong Jiatong Machinery Manufacturing Co., Ltd., Chengde, China was integrated at each tube end during wet filament winding. During winding and cure, the two end inserts were fixed to a steel central shaft mounted on a dedicated tooling frame to maintain nominal axial alignment and concentricity. The dimensions refer to the non-threaded effective/load-transfer section: D31 used a 31/21 mm tube outside/inside diameter and a 21/14 mm insert outside/inside diameter; D33 used 33/23 and 23/16 mm; D35 used 35/25 and 25/18 mm; and the end-enlarged configuration used a 33/23 mm central tube section, 35/25 mm end regions, and a 23/16 mm insert section. The engagement length was 60 mm. The grooves were produced by milling: five longitudinal grooves with a depth of 1 mm, width of 5 mm, and spacing of 6 mm, and seven circumferential grooves with a depth of 1 mm and width of 3 mm, equally distributed around the circumference. The specimen geometry and dimensional definitions are shown in Figure 1. Table S2a summarizes the geometry, Table S2b summarizes the manufacturing parameters, and Figure S1 shows the physical features of the insert. Figure S2 documents the shaft-based alignment arrangement.

The composite tubes were manufactured by wet filament winding using T700 carbon fibers (Beijing Bairuiding Technology Co., Ltd., Beijing, China) and the TDE86 epoxy-resin system (Institute of Petrochemistry, Heilongjiang Academy of Sciences, Harbin, China); J-250 epoxy adhesive (Institute of Petrochemistry, Heilongjiang Academy of Sciences, Harbin, China) was used in the fibe–insert region. The documented equivalent-lamina thickness was 0.15 mm. Positive winding angles denote clockwise winding when viewed along the tube longitudinal direction, negative angles denote counterclockwise winding, and 90° denotes circumferential winding. The sequence [(±30°)_3_/±45°/90°/±45°]_2_/[(±30°)_2_/±45°/(90°)_3_] contains 31 equivalent laminae: eight +30°, eight −30°, five +45°, five −45°, and five 90° laminae. The complete ply-by-ply order is given in Table S3, and the constituent reference properties are listed in Table 1 [33].

Before winding, the external insert surface was sandblasted at 0.4–0.6 MPa using 120–180 mesh white fused alumina for 15–30 s, with a specified roughness range of Ra 2.5–5.0 μm [53,54,55,56]. Sandblasting was performed using a Ah-1010 sandblasting unit (Zhongshan Aihang Machinery Equipment Co., Ltd., Zhongshan, China) with 120–180 mesh white fused alumina supplied by Changlong Jiatong Machinery Manufacturing Co., Ltd., Chengde, China. J-250 was applied manually during winding, and the applied amount was controlled by an experienced operator to maintain a nominal bond-line thickness of 0.1–0.15 mm. The bond-line thickness was not measured specimen by specimen. No separate compaction pressure was applied during winding; circumferential yarns were tightened around the engagement region, and glass cloth was used to absorb excess surface adhesive/resin. The wound assembly was co-cured at 80 °C for 1–2 h. After full cure, the tube ends were trimmed to produce flat end faces. This post-cure finishing operation did not reposition the embedded inserts.

Each ambient equal-section diameter group comprised three specimens tested at 1 mm/min; each post-conditioned equal-section diameter group likewise comprised three specimens tested at 1 mm/min. The ambient and post-conditioned equal-section cohorts came from the same production batch and were manufactured using the same nominal winding, adhesive-application, and curing procedures. The cohorts consisted of independent specimens rather than matched pairs. Potential contributions from adhesive distribution, groove filling, fiber volume fraction, void content, insert alignment, and end-region consolidation are therefore considered sources of experimental variability rather than being analyzed as individually controlled factors. The shaft-and-frame tooling provided nominal insert alignment and concentricity, but residual specimen-specific alignment and concentricity were not measured.

The ambient equal-section specimen identifiers were D31-RT-A to D31-RT-C, D33-RT-A to D33-RT-C, and D35-RT-A to D35-RT-C. D31-RT-A to D31-RT-C are reanalyzed baseline records from the room-temperature D31 program reported by Luo et al. [33]. The D33 and D35 ambient records were not analyzed in that paper. Table S4 summarizes the boundary between the earlier and present analyses. The end-enlarged series comprised three additional ambient specimens, End-enlarged-01 to End-enlarged-03. Unique manuscript identifiers are used throughout, no specimen was reused, and the complete specimen register is provided in Table S5.

### 2.2. Conditioning and Axial Tension

The ambient equal-section series comprised nine specimens: three specimens in each of the D31, D33, and D35 groups. All were tested at 1 mm/min.

**Figure 2:**
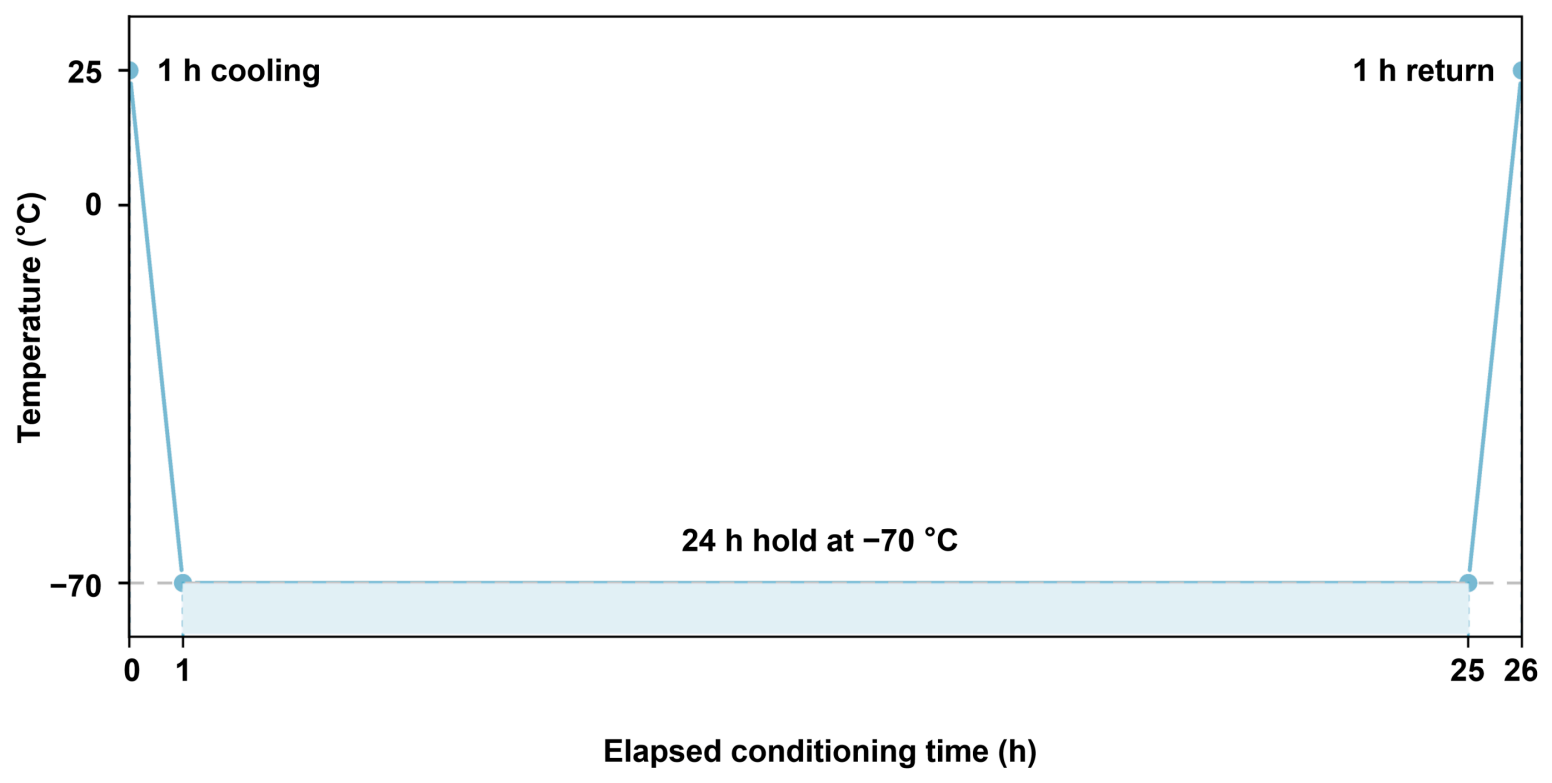
Prescribed low-temperature conditioning cycle before tensile testing. The blue-shaded region denotes the 24 h hold at the −70 °C chamber setpoint; the two 1 h intervals are nominal chamber-setpoint transition times rather than prescribed constant-rate ramps.

The environmental-simulation (ES) specimens were conditioned in a chamber using the programmed setpoint history 25 → −70 → 25 °C: a nominal 1 h setpoint transition from 25 to −70 °C, a 24 h hold at the −70 °C setpoint, and a nominal 1 h setpoint transition back to 25 °C (Figure 2). The two 1 h intervals were nominal chamber-setpoint transition times; the cooling and heating ramps were not controlled at a prescribed rate. No specimen-mounted thermocouple was used; therefore, −70 °C denotes the chamber setpoint rather than a directly measured specimen temperature. After the chamber returned to 25 °C, the specimens were removed and held for 24 h under room conditions before axial testing. Slight condensation was noted after removal, but no obvious macroscopic damage was recorded; the design does not isolate or quantify any residual condensation effect. The reported response is consequently post-conditioning room-temperature behavior, not in-situ −70 °C behavior.

The end-enlarged specimens were tested at room temperature. All axial tensile tests were conducted under displacement control at 1 mm/min using an Instron 8801 servo-hydraulic testing machine with a 250 kN capacity (Instron, Norwood, MA, USA). Load was introduced entirely through custom 42CrMo threaded loading members connected to the two end inserts; the composite tube body was not directly gripped. An M16 threaded loading interface was used for the D31 baseline configuration, whereas the non-threaded effective-section dimensions are configuration-specific (Table S2a). Figure S3 shows the test setup and load path. Force and crosshead/machine displacement were recorded directly by the testing machine, and visible damage was documented with a digital camera.

Machine displacement includes deformation and compliance of the machine, grips, loading members, threaded interfaces, inserts, and composite specimen. System compliance was not measured separately, and local insert–composite slip was not measured. Machine displacement is therefore reported only as a machine-level record and is not interpreted as local joint slip, specimen strain, stiffness, or ductility.

### 2.3. Endpoint Definition and Statistical Analysis

The peak load was defined as the maximum recorded force during monotonic loading. The analysis comprised 21 independent specimens: nine ambient equal-section specimens, nine post-conditioned ES equal-section specimens, and three ambient end-enlarged specimens. Data S1 lists the specimen-level results.

The arithmetic mean, sample standard deviation (SD), coefficient of variation (CV), range, and two-sided 95% confidence interval (CI) of the mean were calculated in OriginPro 2022 (OriginLab Corporation, Northampton, MA, USA). Summary statistics were calculated from the unrounded measurements and are compiled in Data S2; specimen-level values in the tables are rounded for presentation. The CI used Student’s t distribution. Comparisons among the independent unmatched series were descriptive; no equivalence test or causal-effect analysis was specified.

The nominal axial stress in the free composite-tube section, excluding the insert-embedded ends, was calculated as

*σ_tube_*_,*nom*_ =*P*/*A_tube_* = 4*P*/[*π*(*D*_0_^2^ − *D_i_*^2^)] (1)

where *P* is the peak load, *A_tube_* is the nominal annular area, and *D*_0_ and *D_i_* are the nominal outer and inner diameters of the free composite-tube section, respectively. The D31, D33, and D35 groups used 31/21, 33/23, and 35/25 mm, respectively. The end-enlarged specimens were evaluated using the 33/23 mm central tube section. Equation (1) gives a nominal tube-section stress and does not represent the local stress within the embedded joint.

Failure observations were classified specimen by specimen from the test observations and macroscopic photographs using insert extraction, visible adhesive/interfacial separation, delamination/layer separation, fiber/ply damage or debris, and splitting/circumferential cracking. The available images generally cannot distinguish cohesive adhesive failure from metal/adhesive or composite/adhesive debonding; Table S6 therefore preserves this evidence limit rather than assigning an unsupported microscopic interface.

### 2.4. Qualitative Load-Transfer Framework

Let *x* denote the axial position along the 60 mm insert engagement, *p* the effective engagement perimeter, and *t*(*x*) the resultant axial traction transferred by the combined adhesive, groove-filled composite, local contact, and surrounding wound plies. Axial equilibrium alone requires

(2) $$P=p\int_{0}^{L_{e}}t(x)\mathrm{dx}$$

For context, a conventional linearized shear-lag idealization relates the relative axial slip *s* = ui − uc to an effective engagement stiffness per unit area *k_s_* through *t* = *k_s_s*, which gives [43,44,45]

*d*^2^*s*/*dx*^2^ − *β_a_*^2^*s* = 0,*β_a_*^2^ = *pk_s_*[1/(*E_i_A_i_*) + 1/(*E_c_A_c_*)] (3)

Here, *E_i_A_i_* and *E_c_A_c_* are effective axial rigidities of the insert and composite paths. In a conventional bonded-tube problem, Equation (3) indicates elevated transfer demand near engagement boundaries and stiffness transitions. In the present joint, however, *k_s_* would combine adhesive, grooves, co-wound fibers, contact, and evolving damage. Because local slip, bond-line deformation, groove-fill quality, and load-train compliance were not measured, no parameter was identified, and the equation was neither calibrated nor validated.

The observed maximum load is bounded qualitatively by the lowest active component limit state,

*P_u_* = min(*P_adh_*,*P_groove_*,*P_delam_*,*P_ply_*,*P_metal_*_/*thread*_) (4)

**Figure 3:**
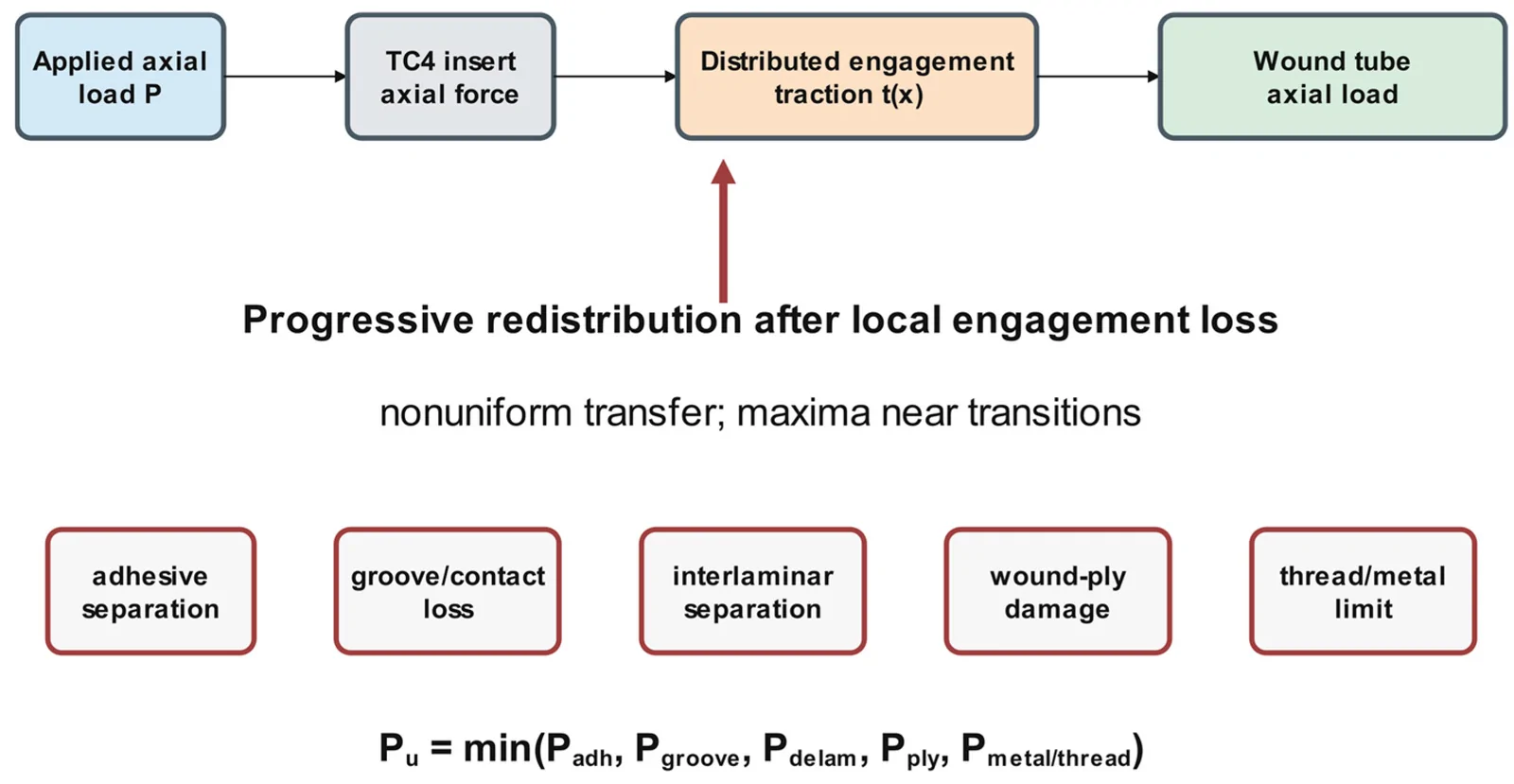
Analytical load-transfer path and competing component limit states.

The terms in Equation (4) denote possible adhesive/interfacial separation, loss of groove engagement, interlaminar separation, wound-ply damage, and failure of the metal/threaded loading path. Equations (2)–(4) and Figure 3 are retained only as a qualitative bookkeeping framework; they are not a predictive model for the present tests.

## 3. Results

### 3.1. Ambient Equal-Section Tensile Response

**Figure 4:**
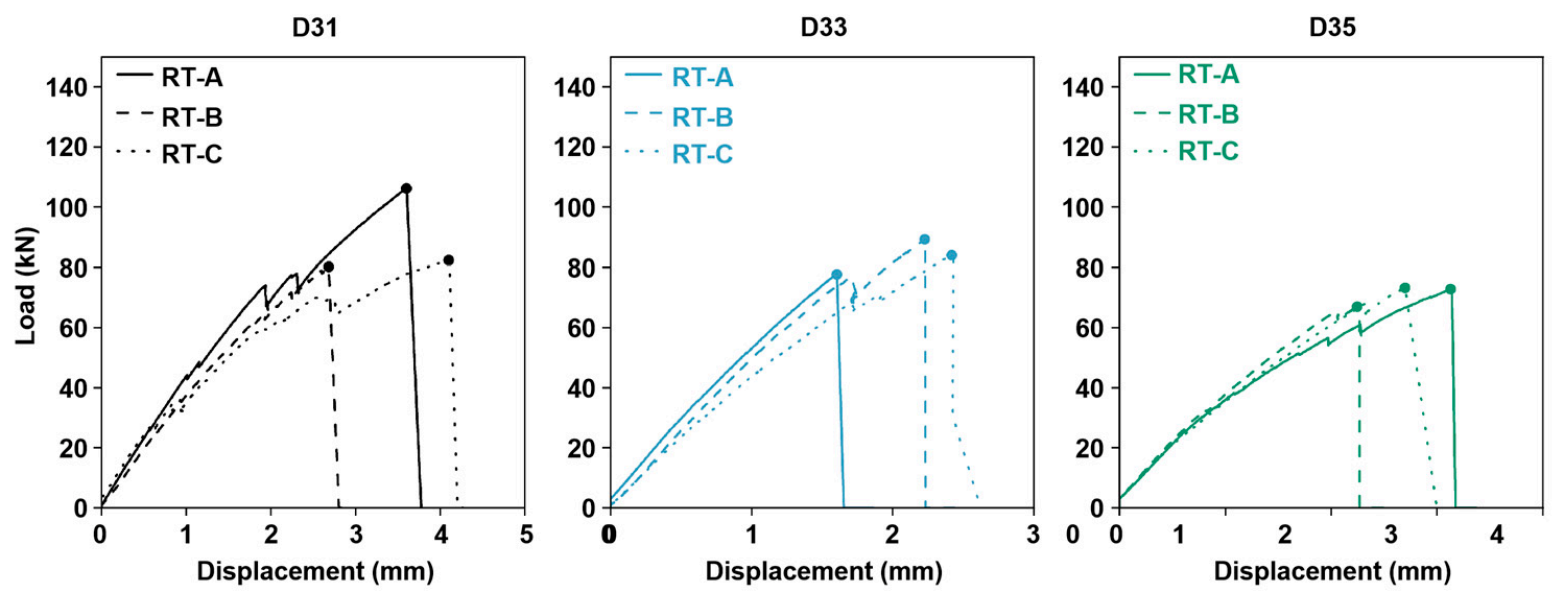
Load–machine-displacement curves of the nine ambient equal-section specimens. Markers indicate peak loads.

The ambient equal-section series contained nine specimens tested at 1 mm/min (Table 2 and Figure 4). The peak loads ranged from 67.023 to 106.160 kN, with a mean of 81.387 kN, an SD of 11.425 kN, a CV of 14.04%, and a 95% CI of 72.605–90.169 kN. The mean nominal tube-section axial stress was 186.676 MPa.

The D31, D33, and D35 ambient groups had mean peak loads of 89.525, 83.613, and 71.023 kN, respectively, and CVs of 16.14%, 6.92%, and 4.89%. Because each diameter group contained only three specimens, and the corresponding CIs were broad, these differences are reported descriptively rather than interpreted as a diameter effect.

### 3.2. Post-Conditioned Equal-Section Response

**Figure 5:**
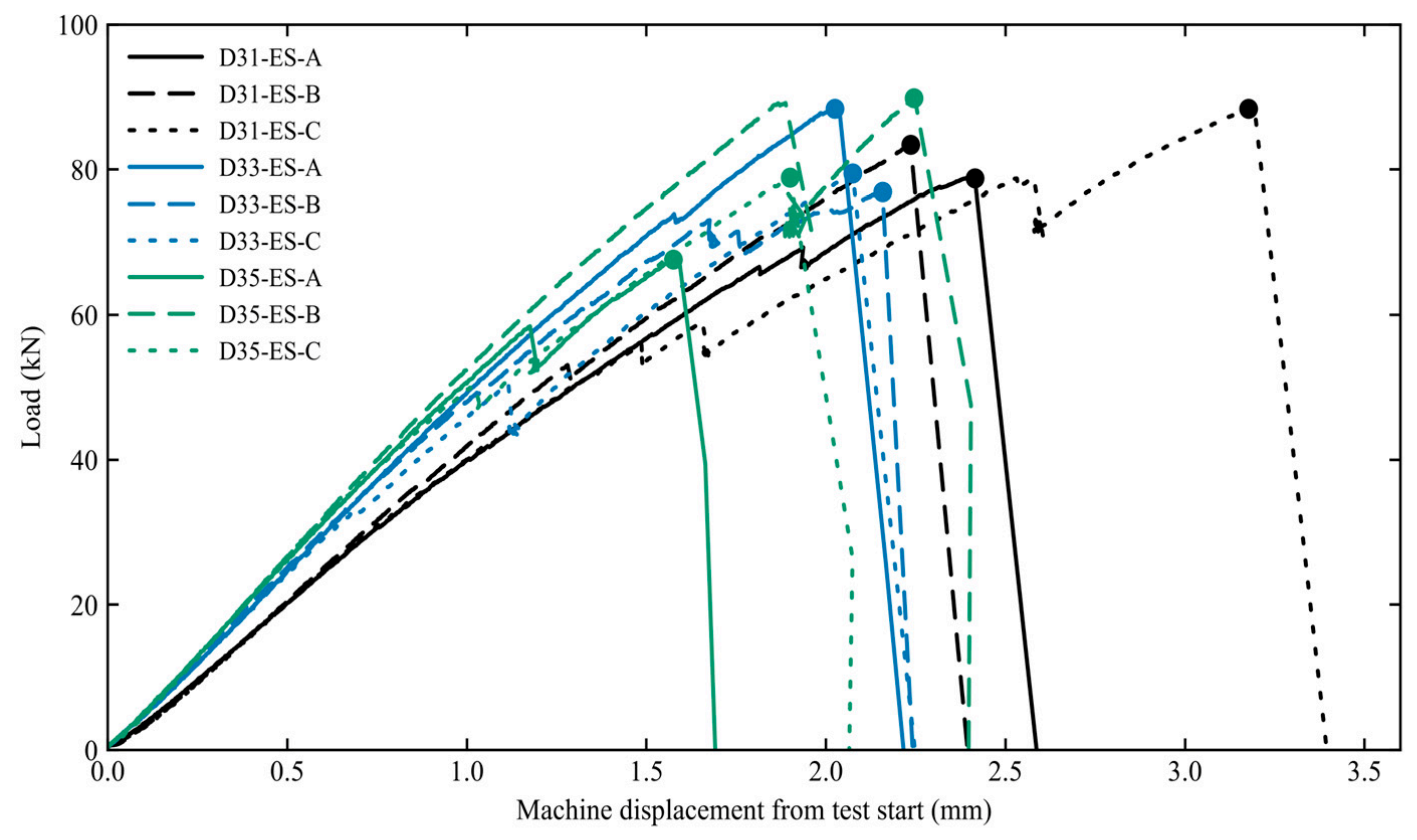
Load–machine-displacement curves of the post-conditioned ES equal-section specimens. Markers indicate peak loads.

The nine post-conditioned ES specimens reached peak loads of 67.620–89.873 kN (Table 3 and Figure 5). The mean peak load was 81.313 kN, with an SD of 7.070 kN, a CV of 8.70%, and a 95% CI of 75.879–86.748 kN. The mean nominal tube-section axial stress was 185.766 MPa.

The D31, D33, and D35 groups had mean peak loads of 83.530, 81.612, and 78.798 kN, respectively, with CVs of 5.73%, 7.38%, and 14.12%. Their broad and overlapping CIs do not show clear separation among the three groups.

The overall mean machine displacement at peak load was 2.20 mm; the D31, D33, and D35 group means were 2.61, 2.09, and 1.91 mm, respectively. These are machine-level coordinates of the load maxima and are not local joint displacements, stiffness values, or ductility measures.

### 3.3. End-Enlarged Response

**Figure 6:**
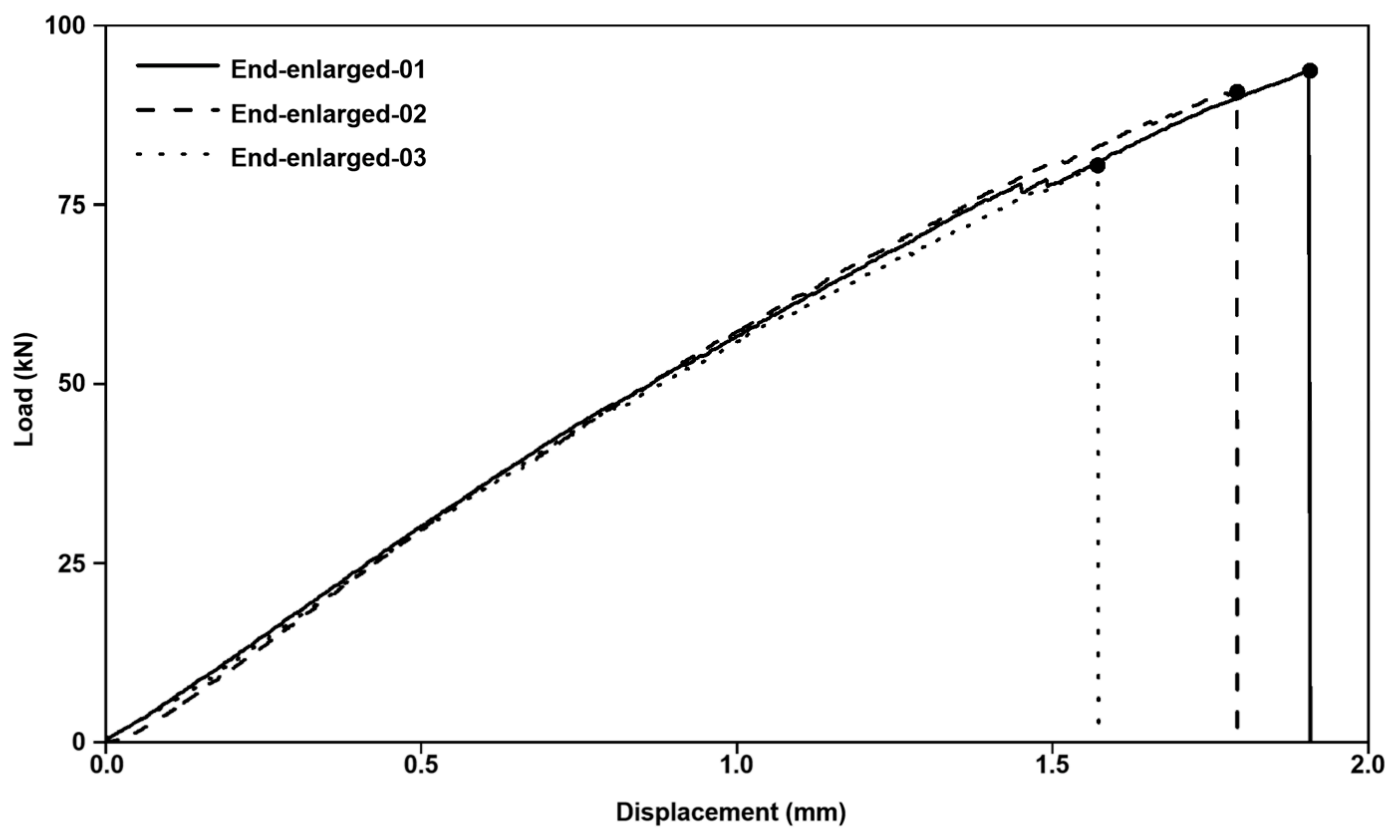
Load–machine-displacement curves of the three end-enlarged specimens. Markers indicate peak loads.

The three end-enlarged specimens reached peak loads of 80.447–93.650 kN (Table 4 and Figure 6). The mean peak load was 88.261 kN, with an SD of 6.927 kN, a CV of 7.85%, and a 95% CI of 71.053–105.468 kN. Based on the 33/23 mm central tube section, the mean nominal axial stress was 200.673 MPa.

As shown in Figure 6, all three curves rose monotonically to their respective peaks and then exhibited abrupt load drops, consistent with the observed pull-out failure family.

### 3.4. Comparison Among Specimen Series

**Figure 7:**
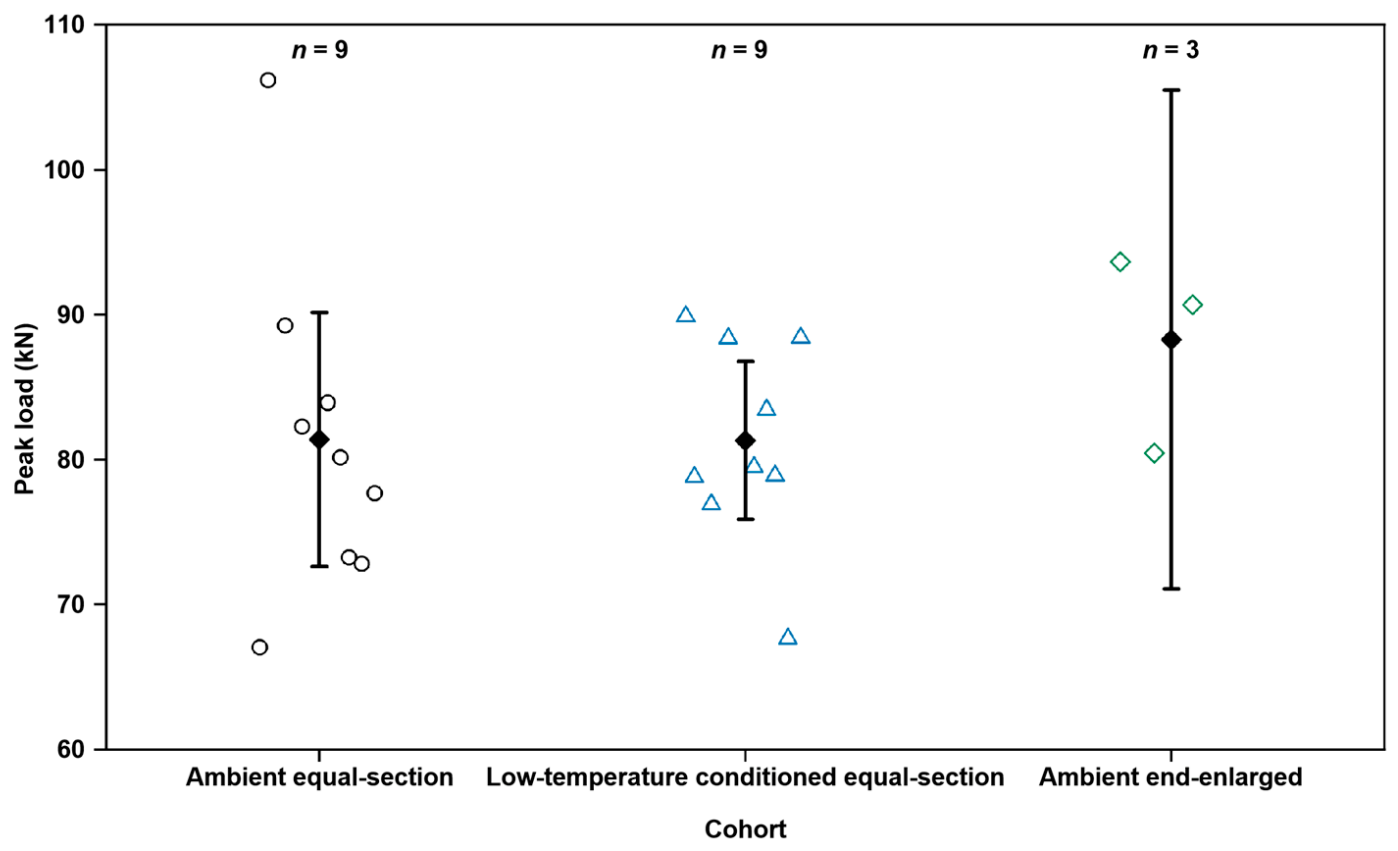
Peak loads of all specimens. Open black circles, open blue triangles, and open green diamonds denote the ambient equal-section, post-conditioned equal-section, and ambient end-enlarged specimens, respectively. Filled black diamonds denote the cohort means, and the error bars indicate two-sided 95% CIs based on Student’s t distribution; *n* denotes the number of specimens in each cohort.

**Figure 8:**
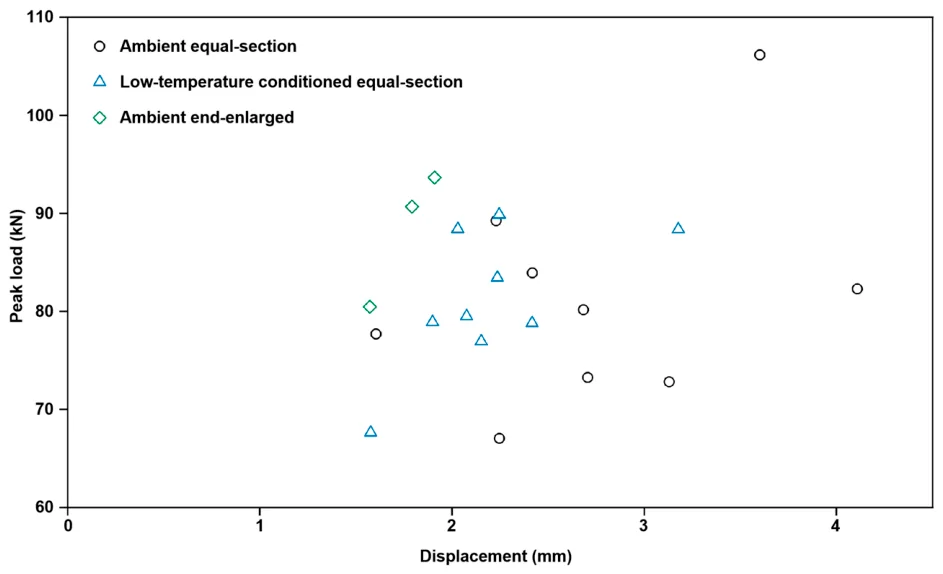
Peak load versus machine displacement at peak load for all 21 specimens.

The individual peak loads and series summaries are compared in Figure 7 and Table 5; Figure 8 relates the peak load to the machine displacement at peak. At 1 mm/min, the post-conditioned and ambient equal-section means were 81.313 and 81.387 kN, respectively, a difference of −0.074 kN (−0.09%); their 95% CIs overlapped. The similarity of these independently sampled cohort means is descriptive and does not demonstrate equivalence, capacity retention, or absence of a conditioning effect.

### 3.5. Recorded Failure Morphology

Across the 21 records, the test observations generally indicated that one insert pulled out first and that later loading sometimes involved the opposite end. Because no synchronized high-speed imaging or local end-displacement record was obtained, abrupt curve drops are treated as the progressive loss of engagement at the specimen level and are not assigned retrospectively to a particular end. Table S6 summarizes the observed event sequence and macroscopic features for every specimen.

**Figure 9:**
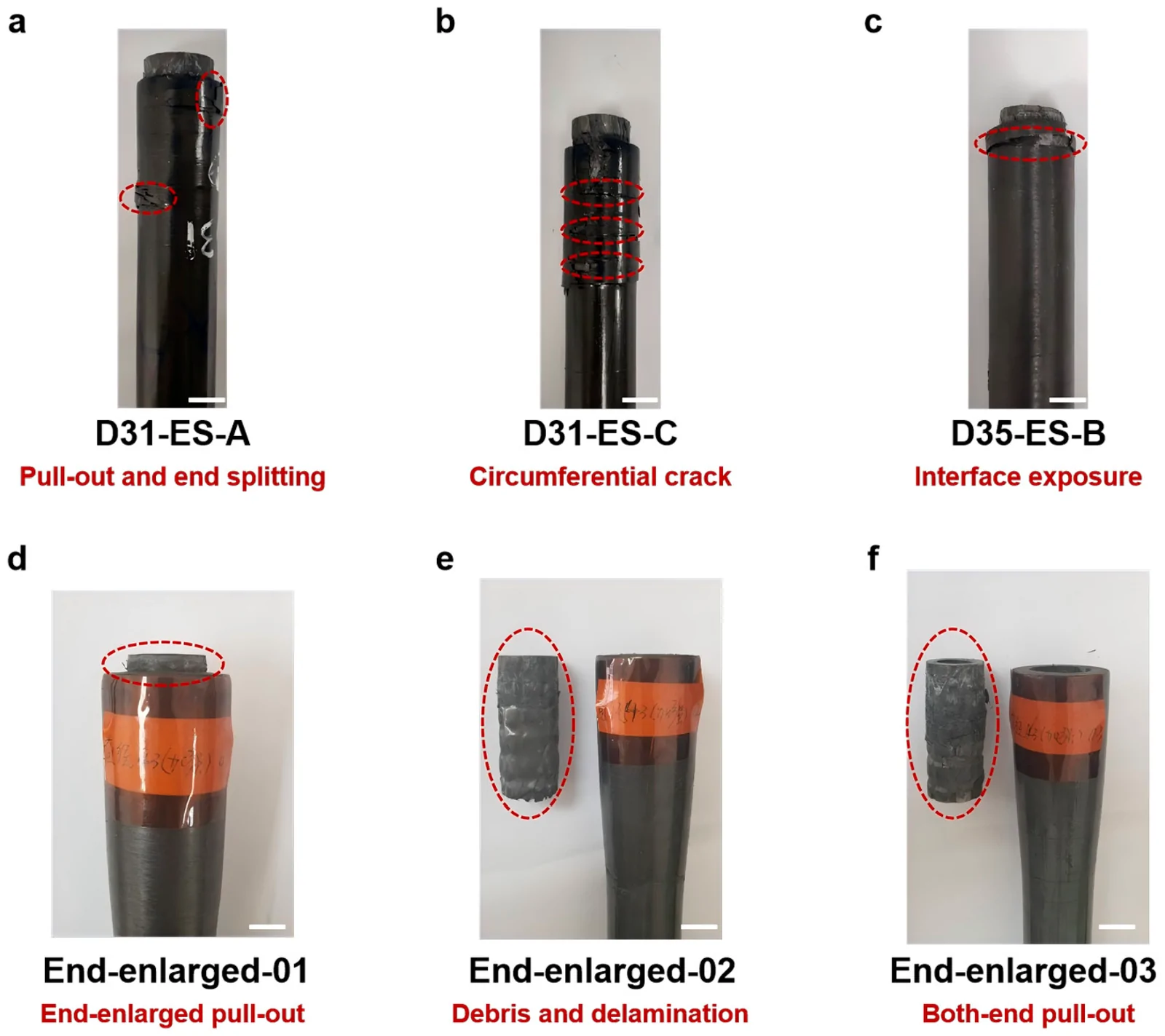
Representative post-test morphologies: (**a**) D31-ES-A, pull-out with end splitting; (**b**) D31-ES-C, circumferential cracking near the insert end; (**c**) D35-ES-B, exposed insert–composite region after pull-out; (**d**) End-enlarged-01, upper-end pull-out; (**e**) End-enlarged-02, debris and delamination; and (**f**) End-enlarged-03, upper-end pull-out with layered fragments and fiber debris. Scale bar, 10 mm. The handwritten markings on the specimen labels are internal specimen identifiers and contain no analytical information.

The post-conditioned specimens repeatedly exhibited extraction of a titanium-alloy insert with adjacent composite material, separation between wound layers, circumferential cracking near an insert end, and axial splitting at a tube end (Figure 9a–c). D31-ES-A showed lower-end pull-out with end splitting, D31-ES-B showed upper-end pull-out and splitting, and D31-ES-C showed circumferential cracking and a comparatively rough extracted surface. The D33 specimens also failed by pull-out and layer separation. D35-ES-A and D35-ES-B showed involvement of both ends in the continued test, whereas D35-ES-C showed lower-end pull-out, while the opposite end remained visually intact.

The end-enlarged specimens likewise showed pull-out-related damage (Figure 9d–f and Figure S4). End-enlarged-01 exhibited upper-end pull-out, End-enlarged-02 showed debris and delamination at the separated region, and End-enlarged-03 showed upper-end pull-out with layered fragments and fiber debris.

## 4. Discussion

### 4.1. Post-Conditioning Room-Temperature Response

At 1 mm/min, the ambient and post-conditioned equal-section series reached mean peak loads of 81.39 and 81.31 kN, respectively. All post-conditioned results fell within the ambient range, and the corresponding CVs were 14.04% and 8.70%. Within these independent unmatched series, the observed difference in the mean peak load was −0.09%. Because no paired design, randomized specimen matching, prespecified equivalence margin, or equivalence test was used, the data neither establish that conditioning had no effect nor quantify the retained capacity.

The corresponding mean nominal tube-section axial stresses were 186.68 and 185.77 MPa. These values normalize the peak load by the nominal free-tube area and do not represent local joint or adhesive strength. The manufacturer reference information lists J-250 bond-strength values at −70 °C of 23 MPa on standard aluminum adherends and 30 MPa on standard copper adherends (Table S7), but these are not specimen-specific constitutive or fracture data for the present TC4/T700–TDE86 co-wound joint. Ref. [35] likewise provides context from a different T700/C204 tubular lap-joint system and thermal protocol but does not validate the present specimen temperature or failure mechanism. Thus, the similar cohort means and visible pull-out family after a chamber cycle plus 24 h at room temperature do not demonstrate in situ −70 °C capacity or isolate adhesive, condensation, residual-stress, and manufacturing contributions.

### 4.2. Mechanics-Based Interpretation of Pull-Out

The qualitative framework indicates that the load transfer need not be uniform over the 60 mm engagement. The local loss of adhesive contact or groove engagement can shift demand to the remaining engaged region and surrounding laminate; so, the final extraction can include adhesive residue, fibers, and layered fragments rather than a clean adhesive surface. This interpretation is consistent with the mixed macroscopic observations, but the machine-level load drops cannot be assigned to a specific groove, interface, or tube end without synchronized local measurements.

The proposed redistribution sequence is also consistent with the finite-element interpretation in Ref. [33], where the adhesive-region damage preceded ±30°-ply damage and interlaminar delamination, and with broader fracture/cohesive-zone studies [46,47,48,49,50]. This agreement is qualitative only: the present equations were not calibrated, local traction/slip was not measured, and the macroscopic photographs do not resolve the microscopic interface.

### 4.3. Influence of End Enlargement and Failure Location

The three end-enlarged specimens reached a mean peak load of 88.26 kN and a CV of 7.85%. Macroscopic failure remained concentrated around the embedded joint. The extracted inserts carried adhesive residue, fibers, and laminate fragments, indicating a mixed visible failure path through the adhesive, groove-filled material, interfaces, and adjacent plies.

Relative to the three ambient D33 equal-section specimens, the end-enlarged specimens had a 4.65 kN (5.56%) higher mean peak load (88.26 versus 83.61 kN); the two 95% CIs were broad and overlapping. Because each cohort contained only three independent, unmatched specimens, this descriptive difference does not isolate a causal effect of increasing the end diameter from 33 to 35 mm. The end transition, insert grooves, adhesive distribution, and local winding sequence should therefore be evaluated as an integrated load-transfer region.

### 4.4. Manufacturing Variability and Design Reliability

The ambient D31, D33, and D35 subgroup means were 89.53, 83.61, and 71.02 kN, whereas the post-conditioned subgroup means were 83.53, 81.61, and 78.80 kN. The stronger apparent ambient decrease with diameter is heavily influenced by D31-RT-A (106.16 kN); D31-RT-B/C alone averaged 81.21 kN. Figure S5 provides the complete D31-RT-A curve, and Table S8 reports the influence analysis. With *n* = 3 per diameter and configuration-specific insert dimensions changing together with the tube diameter, these patterns are exploratory and do not establish a diameter effect. Plausible contributors include curvature and local stiffness, adhesive distribution, groove filling, alignment, and end-region consolidation, none of which was independently varied.

The three principal cohorts had observed CVs of 14.04%, 8.70%, and 7.85%, respectively; the end-enlarged estimate is especially uncertain at *n* = 3. Although the ambient and post-conditioned equal-section specimens came from the same production batch and nominal process specification, the adhesive amount was controlled by operator experience to maintain a nominal bond-line thickness of 0.1–0.15 mm, and the thickness was not measured specimen by specimen. A steel central shaft mounted on a dedicated tooling frame provided nominal insert alignment and concentricity during winding and cure, but residual alignment and concentricity were not quantified for individual specimens. Groove filling, fiber volume fraction, void content, and end-region consolidation were not characterized specimen by specimen [51,52]. These variables remain credible sources of between-specimen and between-cohort variation and limit causal interpretation despite the common nominal process.

### 4.5. Engineering Implications and Limitations

No macroscopic failure was observed in the free composite-tube section, whereas visible failure remained concentrated around the inserts. The environmental assessment comprised one chamber-setpoint cycle, removal after return to 25 °C, and a 24 h room-temperature hold before testing. The experimental program did not include specimen thermocouples, humidity control, local slip or system-compliance measurements, bond-line mapping, internal-damage scanning, or specimen-level dimensional quality measurements. Slight condensation was observed after removal but was not an independently controlled factor. The independent cohorts, small *n* = 3 diameter/end-enlarged subgroups, and D31-RT-A influence further limit precision. Future qualification should use prospectively matched or randomized-batch specimens, a prespecified practically relevant equivalence margin with sample-size planning and formal two-one-sided equivalence tests, repeated and in situ low-temperature loading, specimen thermocouples, controlled transfer/humidity, dimensional and nondestructive inspection, and local deformation measurements.

## 5. Conclusions

This study evaluated the axial tensile behavior of filament-wound T700/TDE86 composite tubes with grooved pre-embedded TC4 titanium-alloy inserts in ambient equal-section, post-conditioned equal-section, and ambient end-enlarged configurations. At 1 mm/min, the three series reached mean peak loads of 81.39, 81.31, and 88.26 kN, respectively, corresponding to mean nominal axial stresses of 186.68, 185.77, and 200.67 MPa in the free composite-tube section.

After one 25 → −70 → 25 °C chamber-setpoint cycle and a subsequent 24 h room-temperature hold, the post-conditioned and ambient equal-section means differed by only −0.07 kN (−0.09%), with CVs of 8.70% and 14.04%, respectively. This similarity is descriptive, because the specimens were independent and unmatched; it does not demonstrate statistical equivalence, absence of a conditioning effect, retained capacity, or in situ low-temperature performance.

The three ambient end-enlarged specimens reached a mean peak load of 88.26 kN and a CV of 7.85%, and their mean was 4.65 kN higher than that of the three ambient D33 specimens. However, the small independent cohorts and their overlapping confidence intervals do not isolate a causal contribution from end enlargement.

Across all series, the visible failure remained concentrated around the embedded joints. Insert pull-out was accompanied by visible adhesive/interfacial separation, interlaminar/layer damage, attached composite fragments, and local splitting. One end generally failed first, but the available machine record and macroscopic observations do not resolve every curve drop or distinguish the microscopic interface.

Taken together, the measured response reflects the integrated effects of insert dimensions and grooves, the adhesive, the wound plies, and the end transition rather than any single feature. The exploratory diameter trend and the three-specimen end-enlarged difference are not causal geometry tests. Further qualification should use matched designs, larger prespecified samples, formal equivalence criteria where relevant, repeated or in situ low-temperature loading, specimen-temperature and humidity control, and direct measurements of the manufacturing quality, local deformation, and internal damage.

## Figures and Tables

**Table 1:** Documented room-temperature reference properties of the constituent system [33].

Material	Elastic Constants	Poisson Ratios	Density (g/cm^3^)	Reported Strength Parameters (MPa)
T700/TDE86 unidirectional ply	*E*_1_ = 130.8 GPa; *E*_2_ = *E*_3_ = 14.0 GPa; *G*_12_ = *G*_31_ = 5.3 GPa; *G*_23_ = 4.7 GPa	*ν*_12_ = *ν*_31_ = 0.26; *ν*_23_ = 0.40	1.59	XT = 1800; XC = 1400; YT = 65; YC = 200; S12 = S13 = 50; S23 = 40
TC4 titanium alloy	*E* = 110 GPa	*ν* = 0.34	4.50	Tensile strength = 1100
J-250 adhesive	*E* = 1.0 GPa	*ν* = 0.30	0.60	Normal strength = 24.5; shear strength = 25

**Table 2:** Ambient equal-section tensile results. D31, D33, and D35 denote the nominal composite-tube outer-diameter groups.

Specimen	Diameter Group	Rate (mm/min)	Peak Load (kN)	Machine Displacement at Peak (mm)	Nominal Tube Axial Stress (MPa)
D31-RT-A	D31	1	106.160	3.602	259.937
D31-RT-B	D31	1	80.138	2.683	196.222
D31-RT-C	D31	1	82.278	4.108	201.461
D33-RT-A	D33	1	77.678	1.604	176.611
D33-RT-B	D33	1	89.244	2.229	202.908
D33-RT-C	D33	1	83.918	2.418	190.800
D35-RT-A	D35	1	72.800	3.130	154.487
D35-RT-B	D35	1	67.023	2.247	142.228
D35-RT-C	D35	1	73.247	2.706	155.434

**Table 3:** Tensile results of the post-conditioned environmental-simulation (ES) equal-section specimens.

Specimen	Diameter Group	Peak Load (kN)	Machine Displacement at Peak (mm)	Nominal Tube Axial Stress (MPa)
D31-ES-A	D31	78.799	2.416	192.943
D31-ES-B	D31	83.427	2.236	204.273
D31-ES-C	D31	88.365	3.177	216.365
D33-ES-A	D33	88.407	2.029	201.006
D33-ES-B	D33	76.932	2.152	174.916
D33-ES-C	D33	79.496	2.075	180.746
D35-ES-A	D35	67.620	1.576	143.493
D35-ES-B	D35	89.873	2.245	190.716
D35-ES-C	D35	78.902	1.898	167.435

**Table 4:** Tensile results of the end-enlarged specimens.

Specimen	Peak Load (kN)	Machine Displacement at Peak (mm)	Nominal Tube Axial Stress (MPa)
End-enlarged-01	93.650	1.909	212.927
End-enlarged-02	90.684	1.792	206.183
End-enlarged-03	80.447	1.573	182.909

**Table 5:** Summary of the tensile results.

Cohort	*n*	Rate (mm/min)	Peak Range (kN)	Mean ± SD (kN)	CV (%)	95% CI of Mean (kN)	Mean Nominal Tube Axial Stress (MPa)
Ambient equal-section	9	1	67.023–106.160	81.387 ± 11.425	14.04	72.605–90.169	186.676
Post-conditioned ES equal-section	9	1	67.620–89.873	81.313 ± 7.070	8.70	75.879–86.748	185.766
Ambient end-enlarged	3	1	80.447–93.650	88.261 ± 6.927	7.85	71.053–105.468	200.673

## Data Availability

The original contributions presented in this study are included in the article/Supplementary Materials. Further inquiries can be directed to the corresponding author.

## Supplementary Materials

The following supporting information can be downloaded at: https://www.mdpi.com/article/10.3390/ma19214526/s1, Figure S1: Physical views of the pre-embedded TC4 insert; Figure S2: Production-stage photograph of the composite tubes and steel central shafts used with the dedicated alignment tooling; Figure S3: Tensile-test setup and load path; Figure S4: Full-frame post-test morphology of End-enlarged-03; Figure S5: Complete D31-RT-A load–machine-displacement record; Table S1: Closest literature, evidence types, and remaining research gap; Table S2a: Configuration-specific tube and insert dimensions; Table S2b: Groove, surface-preparation, winding, cure, conditioning, and test details; Table S3: Complete equivalent-lamina sequence from the first to the thirty-first layer; Table S4: Relationship between the present records and Luo et al. [33]; Table S5: Specimen register and cohort assignment; Table S6: Observed event sequence and visible macroscopic features for every specimen; Table S7: Manufacturer-reported J-250 bond-strength reference values at −70 °C; Table S8: Sensitivity of the ambient summary to D31-RT-A; Data S1: Specimen-level tensile results; Data S2: Cohort summary statistics.

## References

[1] Ravichandran B., Balasubramanian M. (2024). Joining methods for Fiber Reinforced Polymer (FRP) composites—A critical review. Compos. Part A Appl. Sci. Manuf..

[2] Galińska A. (2020). Mechanical Joining of Fibre Reinforced Polymer Composites to Metals—A Review: Part I Bolted Joining. Polymers.

[3] Bodjona K., Lessard L. (2016). Hybrid bonded-fastened joints and their application in composite structures: A general review. J. Reinf. Plast. Compos..

[4] El-Sisi A., Hassanin A., Alsharari F., Galustanian N., Salim H. (2022). Failure Behavior of Composite Bolted Joints: Review. CivilEng.

[5] Gamdani F., Boukhili R., Vadean A. (2019). Tensile behavior of hybrid multi-bolted/bonded joints in composite laminates. Int. J. Adhes. Adhes..

[6] Heimbs S., Schmeer S., Blaurock J., Steeger S. (2013). Static and dynamic failure behaviour of bolted joints in carbon fibre composites. Compos. Part A Appl. Sci. Manuf..

[7] Wei Y., Jin X., Luo Q., Li Q., Sun G. (2024). Adhesively bonded joints—A review on design, manufacturing, experiments, modeling and challenges. Compos. Part B Eng..

[8] Kupski J., Teixeira de Freitas S. (2021). Design of adhesively bonded lap joints with laminated CFRP adherends: Review, challenges and new opportunities for aerospace structures. Compos. Struct..

[9] Oshima S., Koyanagi J. (2025). Review on Damage and Failure in Adhesively Bonded Composite Joints: A Microscopic Aspect. Polymers.

[10] Budzik M.K., Wolfahrt M., Reis P., Kozłowski M., Sena-Cruz J., Papadakis L., Nasr Saleh M., Machalicka K.V., Teixeira de Freitas S., Vassilopoulos A.P. (2021). Testing mechanical performance of adhesively bonded composite joints in engineering applications: An overview. J. Adhes..

[11] Mohabeddine A., Malik G., Correia J., Silva F., De Jesus A., Fantuzzi N., Castro J.M. (2023). Experimental parametric investigation on the behavior of adhesively bonded CFRP/steel joints. Compos. Struct..

[12] Oshima S., Mikami T., Yoshimura A., Hirano Y., Ogasawara T. (2023). In situ optical observation of damage and failure process in adhesively bonded CFRP joints under mixed-mode loading. Compos. Struct..

[13] Hacısalihoglu İ., Akpinar S. (2022). The effect of stepped notches and recesses on joint strength in adhesive bonded joints: Experimental and numerical analysis. Theor. Appl. Fract. Mech..

[14] Zheng G., Liu C., Han X., Li W. (2020). Effect of spew fillet on adhesively bonded single lap joints with CFRP and aluminum-alloy immersed in distilled water. Int. J. Adhes. Adhes..

[15] Wang J., Ding H., Jiang J., Bi Y. (2023). Fracture analysis of composite-titanium adhesively bonded joints under mode-I loading. Eng. Fract. Mech..

[16] Tsokanas P., Loutas T., Nijhuis P. (2020). Interfacial Fracture Toughness Assessment of a New Titanium–CFRP Adhesive Joint: An Experimental Comparative Study. Metals.

[17] Hu Y., Zhang J., Wang L., Jiang H., Cheng F., Hu X. (2022). A simple and effective resin pre-coating treatment on grinded, acid pickled and anodised substrates for stronger adhesive bonding between Ti-6Al-4V titanium alloy and CFRP. Surf. Coat. Technol..

[18] Liu Z., Wang H., Chen Y., Chai D., Hua L. (2023). Interfacial enhancement of CFRP/titanium alloy bonding joint by constructing a 3D interconnected CNTs network. J. Mater. Res. Technol..

[19] Faudree M.C., Uchida H.T., Kimura H., Kaneko S., Salvia M., Nishi Y. (2022). Advances in Titanium/Polymer Hybrid Joints by Carbon Fiber Plug Insert: Current Status and Review. Materials.

[20] Prakash C., Kumar V., Mistri A., Uppal A.S., Babbar A., Pathri B.P., Mago J., Sharma A., Singh S., Wu L.Y. (2021). Investigation of Functionally Graded Adherents on Failure of Socket Joint of FRP Composite Tubes. Materials.

[21] Chang Y., Wen W., Xu Y., Cui H., Xu Y. (2022). Quasi-static mechanical behavior of filament wound composite thin-walled tubes: Tension, torsion, and multi-axial loading. Thin-Walled Struct..

[22] Chang Y., Zhou Y., Wang N., Lu K., Wen W., Xu Y. (2023). Micro-mechanical damage simulation of filament-wound composite with various winding angle under multi-axial loading. Compos. Struct..

[23] Martins L.A.L., Bastian F.L., Netto T.A. (2014). Reviewing some design issues for filament wound composite tubes. Mater. Des..

[24] Mertiny P., Ellyin F. (2002). Influence of the filament winding tension on physical and mechanical properties of reinforced composites. Compos. Part A Appl. Sci. Manuf..

[25] Geng P., Xing J., Wang Q. (2021). Analytical model for stress and deformation of multiple-winding-angle filament-wound composite pipes/vessels under multiple combined loads. Appl. Math. Model..

[26] Reda R., Khamis M., Ragab A.E., Elsayed A., Negm A.M. (2024). Numerical analysis of the impact of winding angles on the mechanical performance of filament wound type 4 composite pressure vessels for compressed hydrogen gas storage. Heliyon.

[27] Ma Q., Rejab M.R.M., Azeem M., Idris M.S., Rani M.F., Kumar A.P. (2023). Axial and radial crushing behaviour of thin-walled carbon fiber-reinforced polymer tubes fabricated by the real-time winding angle measurement system. Forces Mech..

[28] Srebrenkoska S., Kochoski F., Srebrenkoska V., Risteska S., Kotynia R. (2023). Effect of Process Parameters on Thermal and Mechanical Properties of Filament Wound Polymer-Based Composite Pipes. Polymers.

[29] Ghahremani P., Najafabadi M.A., Alimirzaei S., Fotouhi M. (2023). Acoustic Emission-Based Analysis of Damage Mechanisms in Filament Wound Fiber Reinforced Composite Tubes. Sensors.

[30] Bianchi I., Forcellese A., Mancia T., Mignanelli C., Simoncini M., Verdini T. (2024). Effect of Heat-Shrinkable Tape Application on the Mechanical Performance of CFRP Components Obtained by a Filament Winding Process. J. Compos. Sci..

[31] Eggers F., Almeida J.H.S., Lisbôa T.V., Amico S.C. (2021). Creep and Residual Properties of Filament-Wound Composite Rings under Radial Compression in Harsh Environments. Polymers.

[32] Akın Y., Kara M. (2024). Mechanical strength and low-velocity impact behavior of glass fiber reinforced filament wound pipes with different number of layers after hydrothermal aging. J. Compos. Mater..

[33] Luo H., Li X., Li Y., He X., Ye J., Li Z. (2020). Damage properties of pre-embedded connection of carbon fiber wound composite tubes. Mater. Today Commun..

[34] Kaiser I., Zhang C., Tan K.T. (2022). Mechanical behavior and failure mechanisms of CFRP and Titanium tubular adhesive lap joints at extreme temperatures. Compos. Struct..

[35] Luo H., Wang Q., Yang Y., Li T., Wu J., Gong W., Feng H., He X. (2025). Axial Tensile Adhesively Bonded Performance of Carbon Fiber Composite Tubes Under Room-Temperature and Low-Temperature Circulation. Materials.

[36] Oshima S., Kitagawa K., Takeda T., Kumazawa H., Kitazono K. (2023). Effects of freeze–thaw cycles on mode I fracture toughness of adhesively bonded CFRP joints. Adv. Compos. Mater..

[37] Kumazawa H., Motoyama H., Hasunuma S. (2024). Influence of bonded joint configuration on the cryogenic strength of double-lap composite-metal adhesively bonded joints. Adv. Compos. Mater..

[38] Brito C.B.G., Sales R.C.M., Donadon M.V. (2020). Effects of temperature and moisture on the fracture behaviour of composite adhesive joints. Int. J. Adhes. Adhes..

[39] Moazzami M., Ayatollahi M.R., Akhavan-Safar A., Teixeira De Freitas S., Poulis J.A., da Silva L.F.M. (2022). Effect of cyclic aging on mode I fracture energy of dissimilar metal/composite DCB adhesive joints. Eng. Fract. Mech..

[40] Aliheidari N., Ameli A. (2024). Retaining high fracture toughness in aged polymer Composite/Adhesive joints through optimization of plasma surface treatment. Compos. Part A Appl. Sci. Manuf..

[41] Jamshidi Z., Hejazi S.M., Sheikhzadeh M., Alirezazadeh A. (2022). A review on critical aspects of thermal shocks and thermal cycles in fiber reinforced polymer composites. J. Reinf. Plast. Compos..

[42] Han C.L., Wang G.-D., Li N., Wang M., Liu X.l., Ma J.h. (2021). Study on interlaminar performance of CNTs/epoxy film enhanced GFRP under low-temperature cycle. Compos. Struct..

[43] Aimmanee S., Jongrakwattana M. (2023). Stress analysis of adhesive-bonded tubular-coupler joints with optimum-stiffness-composite adherend under axial tension, pressure, and thermal loading. Int. J. Adhes. Adhes..

[44] Lubkin J.L., Reissner E. (1956). Stress Distribution and Design Data for Adhesive Lap Joints Between Circular Tubes. J. Fluids Eng..

[45] Adams R.D., Peppiatt N.A. (2006). Stress Analysis of Adhesive Bonded Tubular Lap Joints. J. Adhes..

[46] Dionísio J.M.M., Ramalho L.D.C., Sánchez-Arce I.J., Campilho R.D.S.G., Belinha J. (2021). Fracture mechanics approach to stress singularity in adhesive joints. Int. J. Fract..

[47] Katsivalis I., Thomsen O.T., Feih S., Achintha M. (2020). Development of cohesive zone models for the prediction of damage and failure of glass/steel adhesive joints. Int. J. Adhes. Adhes..

[48] Moazzami M., Akhavan-Safar A., Ayatollahi M.R., Poulis J.A., da Silva L.F.M., Teixeira De Freitas S. (2023). A degradable mode I cohesive zone model developed for damage and fracture analysis of dissimilar composite/metal adhesive joints subjected to cyclic ageing conditions. Theor. Appl. Fract. Mech..

[49] Oshima S., Kobayashi S. (2024). Experimental and numerical investigation of mesoscopic damage accumulation in adhesively bonded composite scarf joints. Compos. Part A Appl. Sci. Manuf..

[50] Wei Y., Luo Q., Li Q., Sun G. (2023). On adhesively bonded joints with a mixed failure mode—An experimental and numerical study. Thin-Walled Struct..

[51] Larson R., Bergan A., Leone F., Kravchenko O.G. (2023). Influence of stochastic adhesive porosity and material variability on failure behavior of adhesively bonded composite sandwich joints. Compos. Struct..

[52] Wu C., Yu Y.-Z., Tam L.-h., He L. (2023). Effects of bondline defects on the bond behaviour of CFRP-steel double strap joints. Compos. Struct..

[53] Zhang D., Huang Y. (2021). Influence of surface roughness and bondline thickness on the bonding performance of epoxy adhesive joints on mild steel substrates. Prog. Org. Coat..

[54] Wang F., Bu H., Ma W., Zhang P., Zhan X. (2022). Influence of the different surface treatments on fracture property of CFRP adhesive joint. J. Adhes. Sci. Technol..

[55] Akman E., Bora M.Ö., Çoban O., Oztoprak B.G. (2021). Laser-induced groove optimization for Al/CFRP adhesive joint strength. Int. J. Adhes. Adhes..

[56] Ramaswamy K., O’Higgins R.M., Kadiyala A.K., McCarthy M.A., McCarthy C.T. (2020). Evaluation of grit-blasting as a pre-treatment for carbon-fibre thermoplastic composite to aluminium bonded joints tested at static and dynamic loading rates. Compos. Part B Eng..

