# Supplementary material for: Tensile Behavior of Pre-Embedded Titanium-Alloy Joints in Filament-Wound Carbon-Fiber Composite Tubes After Low-Temperature Conditioning
Source: Materials (Basel). 2026 Oct 27;19(21):4526. doi: 10.3390/ma19214526 (PMC13764296; doi:10.3390/ma19214526)
Supplement: Supplementary file 1 [file materials-19-04526-s001.zip › materials-4608333-supplementary.pdf]

Supporting Information

Tensile behavior of pre-embedded titanium-alloy joints in filament-wound carbon-fiber composite tubes after low-temperature conditioning

Xiaoyong Liu, Zifei Ma, Siyuan Li, Cheng Guo, Haibo Luo, and Jun Wu*

Contents

Table S1 to S8

Data S1 to S2

Figure S1 to S5

Table S1. Closest literature, evidence types, and remaining research gap.

The comparison separates test temperature from conditioning history so that in-situ low-temperature loading is not conflated with room-temperature testing after conditioning.

Study	Joint/material/evidence	Thermal history and test temperature	What it establishes	Why it does not answer the present question
Luo et al. [33]	Same T700/TDE86 tube family; grooved co-wound TC4 insert; D31 experiments and FE interpretation	Ambient; room-temperature loading	Baseline feasibility and proposed adhesive-region → ±30° ply → interlaminar sequence	No D33/D35 ambient analysis, post-conditioned cohort, or end-enlarged cohort
Kaiser et al. [34]	Conventional thin-walled CFRP/Ti tubular adhesive lap joints	In-situ cold, room, and elevated temperatures	Temperature and bond length can alter capacity and competing modes	Different lap-joint architecture and in-situ test-temperature question
Luo et al. [35]	Adhesively bonded T700/C204 composite and TC4 tubes; different resin, dimensions, J-250 film construction, cure/pressure schedule	Room temperature, in-situ −65 °C, and room–low–room history; 24 h room stabilization in the cycle protocol	Thermal history/test temperature can change capacity and failure mode	Does not establish the temperature or mechanism of the present co-wound insert; protocol and materials differ
Oshima et al. [36]; Kumazawa et al. [37]	Bonded CFRP coupon/double-lap composite–metal configurations	Freeze–thaw cycling or cryogenic/room loading	Direct mechanism evidence for cycling, mismatch, and configuration sensitivity	Mechanism-relevant but not component-level validation of this joint
Refs. [38–42]	Bonded-joint and laminate studies under temperature, moisture, aging, or cycling	Heterogeneous protocols	Plausible environmental sensitivity of adhesive/matrix/interlaminar behavior	Cannot define an effect size for the present joint
Refs. [43–50]	Analytical, fracture, cohesive-zone, FE, discrete-element, and in-situ damage studies	Mostly model/mechanism studies	Supports nonuniform transfer and coupled damage	Local slip, bond-line deformation, groove fill, and compliance are unavailable here; no quantitative calibration
Present study	Grooved TC4 inserts co-wound into T700/TDE86 tubes; 21 destructive records	Nine ambient; nine tested at room temperature after 25 → −70 → 25 °C setpoint cycle plus 24 h room hold; three ambient end-enlarged	Peak-load distributions, machine-level histories, nominal tube stress, and macroscopic failure family	Independent/unmatched small cohorts; no in-situ −70 °C strength, equivalence, repeated-cycle durability, or causal geometry effect

Resulting gap. The closest literature does not report the room-temperature axial response of an independently manufactured cohort of this grooved, co-wound TC4/T700–TDE86 joint after the specified chamber cycle and 24 h room-temperature hold, nor the descriptive response of the three ambient end-enlarged specimens.

Table S2a. Configuration-specific tube and insert dimensions.

“Equal-section” means that the nominal composite-tube section is constant along the shell/gauge

region. The insert dimensions in Table S2a refer to the non-threaded effective/load-transfer section and are distinct from the threaded loading interface.

Configuration	Composite-tube section, OD/ID (mm)	Non-threaded insert section, OD/ID (mm)	Embedded-end tube section, OD/ID (mm)	Engagement length (mm)
D31 equal-section	31/21	21/14	31/21	60
D33 equal-section	33/23	23/16	33/23	60
D35 equal-section	35/25	25/18	35/25	60
End-enlarged	Central section: 33/23	23/16	35/25 at both ends	60

Table S2b. Groove, surface-preparation, winding, cure, conditioning, and test details.

Item	Value or procedure used	Scope or interpretation
Longitudinal grooves	Milled; depth 1 mm; width 5 mm; spacing 6 mm; number 5	Non-threaded load-transfer region
Circumferential grooves	Milled; depth 1 mm; width 3 mm; number 7; equally distributed around the circumference	Non-threaded load-transfer region
Insert material	TC4 titanium alloy	Pre-embedded load-transfer insert
Composite system	T700 carbon fiber / TDE86 epoxy; wet filament winding	Constituent reference properties are listed in manuscript Table 1 [33]
Adhesive	J-250 epoxy adhesive applied manually during winding; nominal bond-line thickness 0.1–0.15 mm	Applied amount controlled by operator experience; thickness not measured specimen by specimen
Insert alignment	Two end inserts fixed to a steel central shaft mounted on a dedicated tooling frame during winding and cure	Tooling maintained nominal axial alignment and concentricity; no specimen-specific alignment or concentricity measurements were recorded
Sandblasting	0.4–0.6 MPa; 120–180 mesh white fused alumina; 15–30 s; specified Ra range of 2.5–5.0 μm	Ra denotes the specified process range
Compaction	No separate compaction pressure applied during winding	Circumferential yarns tightened around the engagement region
Outer treatment	Glass cloth used to absorb excess surface adhesive/resin	Applied during winding
Cure	80 °C for 1–2 h	Co-curing of the wound assembly
End finishing	Tube ends trimmed after full cure to obtain flat faces	Post-cure finishing did not reposition the embedded inserts
Manufacturing batch	Ambient and post-conditioned equal-section specimens from the same production batch and nominal process	Independent specimens; no specimen was reused
Conditioning	Chamber setpoint 25 → −70 °C over a nominal 1 h transition; hold 24 h; return to 25 °C over a nominal 1 h transition; remove; hold 24 h at room temperature; then test	The 1 h values are nominal chamber-setpoint transition times, not prescribed constant-rate ramps; no specimen thermocouple was used; slight condensation was observed after removal
Axial test	Instron 8801, 250 kN; displacement control; 1 mm/min	Load introduced through both threaded inserts; tube body not directly gripped
Displacement	Machine/crosshead displacement	Machine-level record including load-train compliance; not local slip, strain, stiffness, or ductility

Table S3. Complete equivalent-lamina sequence from the first to the thirty-first layer.

Positive angles denote clockwise winding when viewed along the tube longitudinal direction,

negative angles denote counterclockwise winding, and 90° denotes circumferential winding. The 31 equivalent laminae comprise eight +30°, eight −30°, five +45°, five −45°, and five 90° laminae.

Layer	Orientation	Layer	Orientation
1	+30°	17	−30°
2	−30°	18	+45°
3	+30°	19	−45°
4	−30°	20	90°
5	+30°	21	+45°
6	−30°	22	−45°
7	+45°	23	+30°
8	−45°	24	−30°
9	90°	25	+30°
10	+45°	26	−30°
11	−45°	27	+45°
12	+30°	28	−45°
13	−30°	29	90°
14	+30°	30	90°
15	−30°	31	90°
16	+30°	—	—

Table S4. Relationship between the present records and Luo et al. [33].

Present records (n)	Relationship to Ref. [31]	Treatment in this revision
D31-RT-A to D31-RT-C (3)	Reanalyzed baseline records from the earlier room-temperature D31 program	Disclosed in the Abstract and Methods; retained as the baseline subset
D33-RT-A to D35-RT-C (6)	Not analyzed in Ref. [31]	Additional ambient records
D31-ES-A to D35-ES-C (9)	Not analyzed in Ref. [31]	Independent ES cohort tested at room temperature after conditioning and a 24 h room-temperature hold
End-enlarged-01 to End-enlarged-03 (3)	Not analyzed in Ref. [31]	Ambient descriptive geometry cohort

Table S5. Specimen register and cohort assignment.

Each identifier in the manuscript denotes one unique destructive-test specimen. The ambient, post-conditioned, and end-enlarged cohorts are independent, and no physical specimen was reused.

Specimen ID	Cohort	Diameter/geometry group	Specimen status
D31-RT-A	Ambient equal-section	D31	Unique destructive specimen; not reused
D31-RT-B	Ambient equal-section	D31	Unique destructive specimen; not reused
D31-RT-C	Ambient equal-section	D31	Unique destructive specimen; not reused
D33-RT-A	Ambient equal-section	D33	Unique destructive specimen; not reused

Specimen ID	Cohort	Diameter/geometry group	Specimen status
D33-RT-B	Ambient equal-section	D33	Unique destructive specimen; not reused
D33-RT-C	Ambient equal-section	D33	Unique destructive specimen; not reused
D35-RT-A	Ambient equal-section	D35	Unique destructive specimen; not reused
D35-RT-B	Ambient equal-section	D35	Unique destructive specimen; not reused
D35-RT-C	Ambient equal-section	D35	Unique destructive specimen; not reused
D31-ES-A	Post-conditioned ES equal-section	D31	Unique destructive specimen; not reused
D31-ES-B	Post-conditioned ES equal-section	D31	Unique destructive specimen; not reused
D31-ES-C	Post-conditioned ES equal-section	D31	Unique destructive specimen; not reused
D33-ES-A	Post-conditioned ES equal-section	D33	Unique destructive specimen; not reused
D33-ES-B	Post-conditioned ES equal-section	D33	Unique destructive specimen; not reused
D33-ES-C	Post-conditioned ES equal-section	D33	Unique destructive specimen; not reused
D35-ES-A	Post-conditioned ES equal-section	D35	Unique destructive specimen; not reused
D35-ES-B	Post-conditioned ES equal-section	D35	Unique destructive specimen; not reused
D35-ES-C	Post-conditioned ES equal-section	D35	Unique destructive specimen; not reused
End-enlarged-01	Ambient end-enlarged	D33 central section / D35 end regions	Unique destructive specimen; not reused
End-enlarged-02	Ambient end-enlarged	D33 central section / D35 end regions	Unique destructive specimen; not reused
End-enlarged-03	Ambient end-enlarged	D33 central section / D35 end regions	Unique destructive specimen; not reused

Table S6. Observed event sequence and visible macroscopic features for every specimen.

Codes: AI, visible adhesive/interfacial separation; DL, delamination/layer separation; FP, fiber/ply damage or debris; SC, splitting or circumferential cracking. Macroscopic evidence generally cannot distinguish cohesive adhesive failure from metal/adhesive or composite/adhesive debonding. The end sequence is stated only to the resolution supported by the test observations.

Specimen ID	Observed event sequence	Visible macroscopic features	Evidence boundary
D31-RT-A	Upper-end extraction; opposite end	AI, DL, FP, and SC at both ends; upper axial	Sequential end involvement

Specimen ID	Observed event sequence	Visible macroscopic features	Evidence boundary
	involved during continued loading	split	
D31-RT-B	Lower-end extraction	AI, DL, FP; slight lower split	Opposite end visually intact
D31-RT-C	Both ends involved; first-end order unresolved	AI, DL, FP; upper split	No synchronized end-resolved record
D33-RT-A	Lower-end followed by upper-end extraction	AI, DL, FP	Sequential end involvement
D33-RT-B	Upper-end cracking followed by lower-end extraction	AI, DL, FP; lower split; upper circumferential crack	Sequential end involvement
D33-RT-C	Lower-end extraction	AI, DL, FP; lower split	Opposite end visually intact
D35-RT-A	Upper-end followed by lower-end extraction	AI, DL, FP; deep lower circumferential fracture	Sequential end involvement
D35-RT-B	Upper-end followed by lower-end extraction	AI, DL, FP; lower split	Sequential end involvement
D35-RT-C	Lower-end followed by upper-end extraction	AI, DL, FP; slight upper split	Sequential end involvement
D31-ES-A	Lower-end extraction	AI, DL, FP; lower split	Opposite end visually intact
D31-ES-B	Upper-end extraction	AI, DL, FP; upper split	Opposite end visually intact
D31-ES-C	Upper-end extraction; later lower-end cracking	Rough AI surface, multiple DL, FP, and SC	Sequential end involvement
D33-ES-A	Upper-end extraction	AI, DL, FP; upper split	Opposite end visually intact
D33-ES-B	Lower-end extraction	AI, DL, FP	Opposite end visually intact
D33-ES-C	Both ends involved; first-end order unresolved	AI, DL, FP; slight lower split	No synchronized end-resolved record
D35-ES-A	Upper-end followed by lower-end extraction	AI, DL, FP	Sequential end involvement
D35-ES-B	Upper-end followed by lower-end involvement during continued loading	AI, DL, FP; upper split	Both ends ultimately involved
D35-ES-C	Lower-end extraction	AI, DL, FP	Opposite end visually intact
End-enlarged-01	Upper-end extraction	AI, DL, FP	Opposite end visually intact
End-enlarged-02	Upper-end extraction	AI, DL, FP/debris	Opposite end visually intact
End-enlarged-03	Upper-end extraction	AI, layered fragments, and FP debris	Opposite end visually intact

Table S7. Manufacturer-reported J-250 bond-strength reference values at −70 °C.

Temperature	Standard adherend	Bond strength (MPa)	Scope
−70 °C	Aluminum	23	Manufacturer reference value; not a property of the present joint
−70 °C	Copper	30	Manufacturer reference value; not a property of the present joint

These values provide material-selection context only. They are not constitutive or fracture parameters for the present TC4/T700–TDE86 co-wound joint and were not used to calculate its load capacity.

Table S8. Sensitivity of the ambient summary to D31-RT-A.

The complete curve reaches 106.160 kN, with pronounced intermediate load drops at approximately 75–80 kN. Because the peak agrees with the recorded maximum and there is no independent basis for excluding the test, D31-RT-A is retained in the primary analysis and its influence is evaluated separately.

Dataset	*n*	Mean ± SD (kN)	CV (%)	Interpretation
All ambient records	9	81.387 ± 11.425	14.04	Primary data set; D31-RT-A retained
Excluding D31-RT-A	8	78.291 ± 7.110	9.08	Influence analysis only; primary analysis unchanged
D31-RT-B/C only	2	81.208 ± 1.513	1.86	Context only; not a replacement D31 estimate

Data S1. Specimen-level tensile results; all tests used a displacement rate of 1 mm/min.

Manuscript ID	Cohort / tube	Peak load (kN)	Machine displacement at peak (mm)	Nominal tube axial stress (MPa)
D31-RT-A	Ambient equal-section / D31	106.160	3.602	259.937
D31-RT-B	Ambient equal-section / D31	80.138	2.683	196.222
D31-RT-C	Ambient equal-section / D31	82.278	4.108	201.461
D33-RT-A	Ambient equal-section / D33	77.678	1.604	176.611
D33-RT-B	Ambient equal-section / D33	89.244	2.229	202.908
D33-RT-C	Ambient equal-section / D33	83.918	2.418	190.800
D35-RT-A	Ambient equal-section / D35	72.800	3.130	154.487
D35-RT-B	Ambient equal-section / D35	67.023	2.247	142.228
D35-RT-C	Ambient equal-section / D35	73.247	2.706	155.434
D31-ES-A	Post-conditioned ES / D31	78.799	2.416	192.943
D31-ES-B	Post-conditioned ES / D31	83.427	2.236	204.273
D31-ES-C	Post-conditioned ES / D31	88.365	3.177	216.365
D33-ES-A	Post-conditioned ES / D33	88.407	2.029	201.006
D33-ES-B	Post-conditioned ES / D33	76.932	2.152	174.916
D33-ES-C	Post-conditioned ES / D33	79.496	2.075	180.746
D35-ES-A	Post-conditioned ES / D35	67.620	1.576	143.493
D35-ES-B	Post-conditioned ES / D35	89.873	2.245	190.716
D35-ES-C	Post-conditioned ES / D35	78.902	1.898	167.435
End-enlarged-01	Ambient end-enlarged / D33 central section / D35 end regions	93.650	1.909	212.927
End-enlarged-02	Ambient end-enlarged / D33 central section / D35 end regions	90.684	1.792	206.183
End-enlarged-03	Ambient end-enlarged / D33 central section / D35 end regions	80.447	1.573	182.909

Machine displacement includes load-train compliance and is not interpreted as local joint displacement, specimen strain, stiffness, or ductility. End-enlarged nominal stress uses the 33/23 mm

central section.

Data S2. Cohort summary statistics.

Cohort	*n*	Peak-load statistics	95% CI and mean nominal stress
Ambient equal-section	9	67.023–106.160 kN; 81.387 ± 11.425 kN; CV 14.04%	CI 72.605–90.169 kN; mean stress 186.676 MPa
Post-conditioned ES	9	67.620–89.873 kN; 81.313 ± 7.070 kN; CV 8.70%	CI 75.879–86.748 kN; mean stress 185.766 MPa
Ambient end-enlarged	3	80.447–93.650 kN; 88.261 ± 6.927 kN; CV 7.85%	CI 71.053–105.468 kN; mean stress 200.673 MPa

The cohorts are independent and unmatched. Confidence-interval overlap does not demonstrate equivalence, and the end-enlarged comparison does not isolate a causal geometry effect.

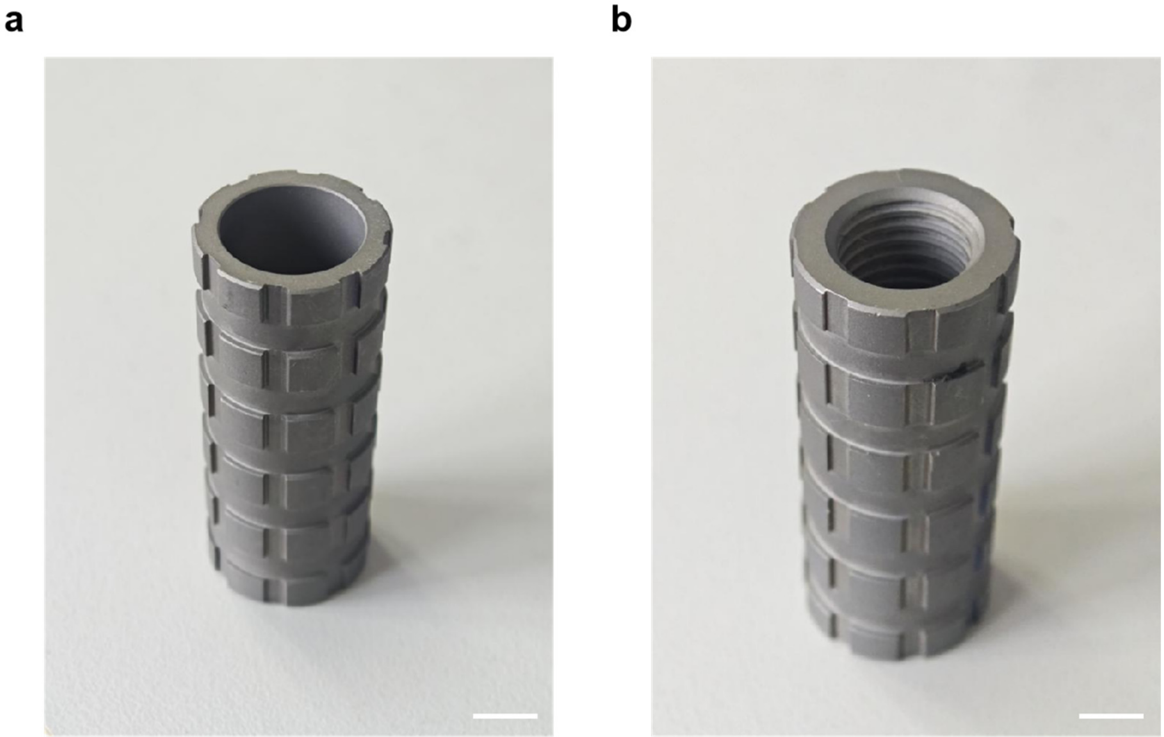

Figure S1. Physical views of the pre-embedded TC4 insert. (a) Non-threaded effective/grooved load-transfer section and (b) threaded loading end. The photographs show the physical features; configuration-specific dimensions are given in Table S2a. Scale bars, 10 mm in a and b.

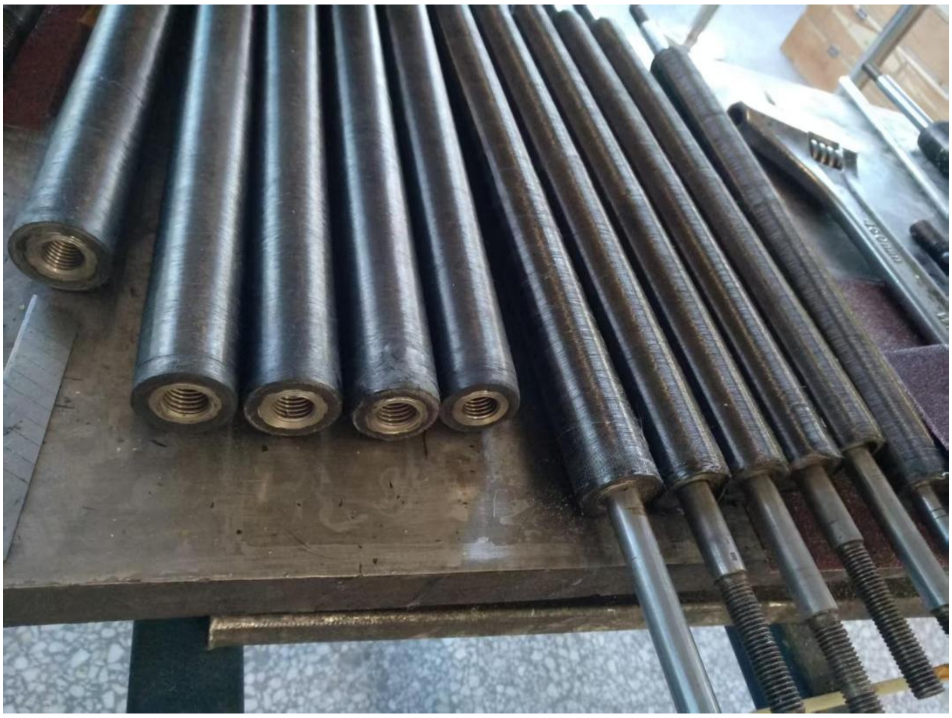

Figure S2. Production-stage photograph of the composite tubes and steel central shafts used with the dedicated alignment tooling. During winding and cure, the two end inserts were fixed to a central shaft mounted on a dedicated tooling frame to maintain nominal axial alignment and concentricity. Residual alignment and concentricity were not measured for individual specimens.

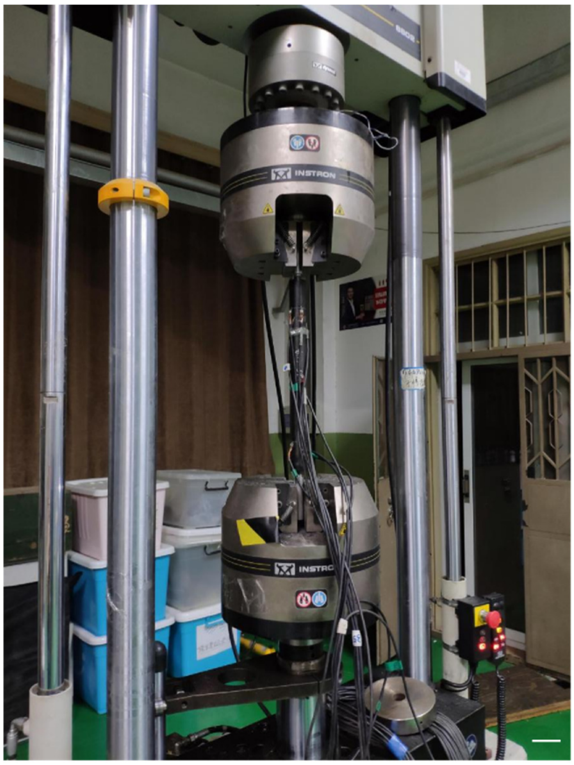

Figure S3. Tensile-test setup and load path. Load path: machine grip → custom 42CrMo loading member → threaded insert connection → co-wound insert/adhesive/composite engagement region → composite tube → opposite insert/loading member → opposite grip. The composite tube body was not directly gripped. Scale bar, 40 mm.

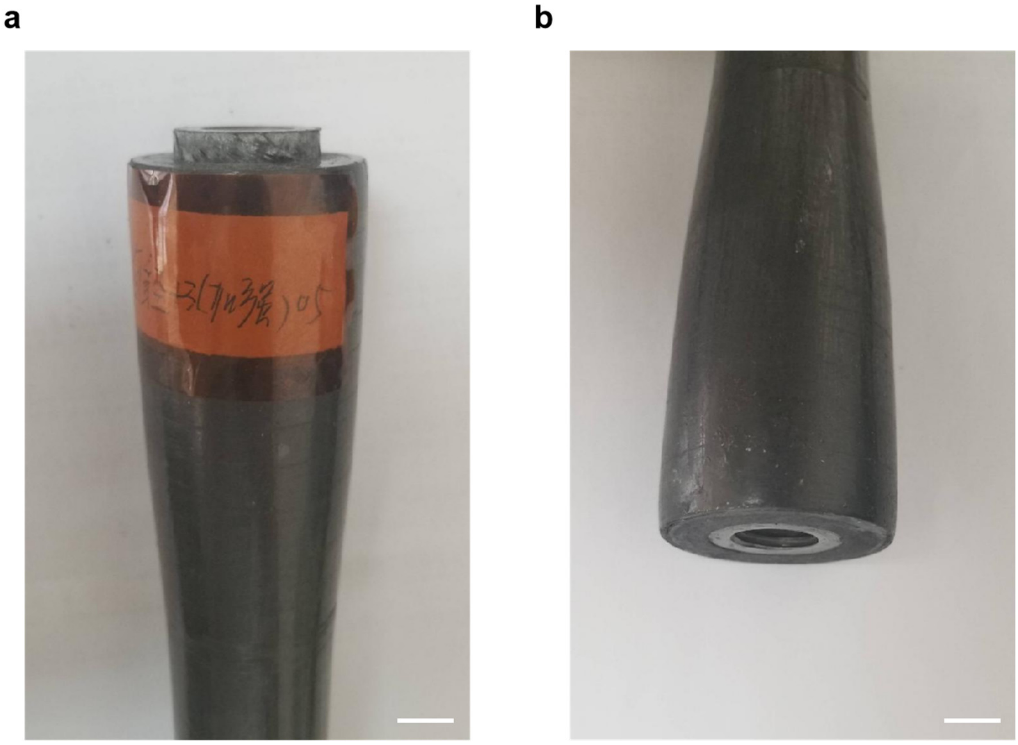

Figure S4. Full-frame post-test morphology of End-enlarged-03. The specimen exhibited upper-end pull-out with layered fragments and fiber debris. Scale bars, 10 mm in a and b.

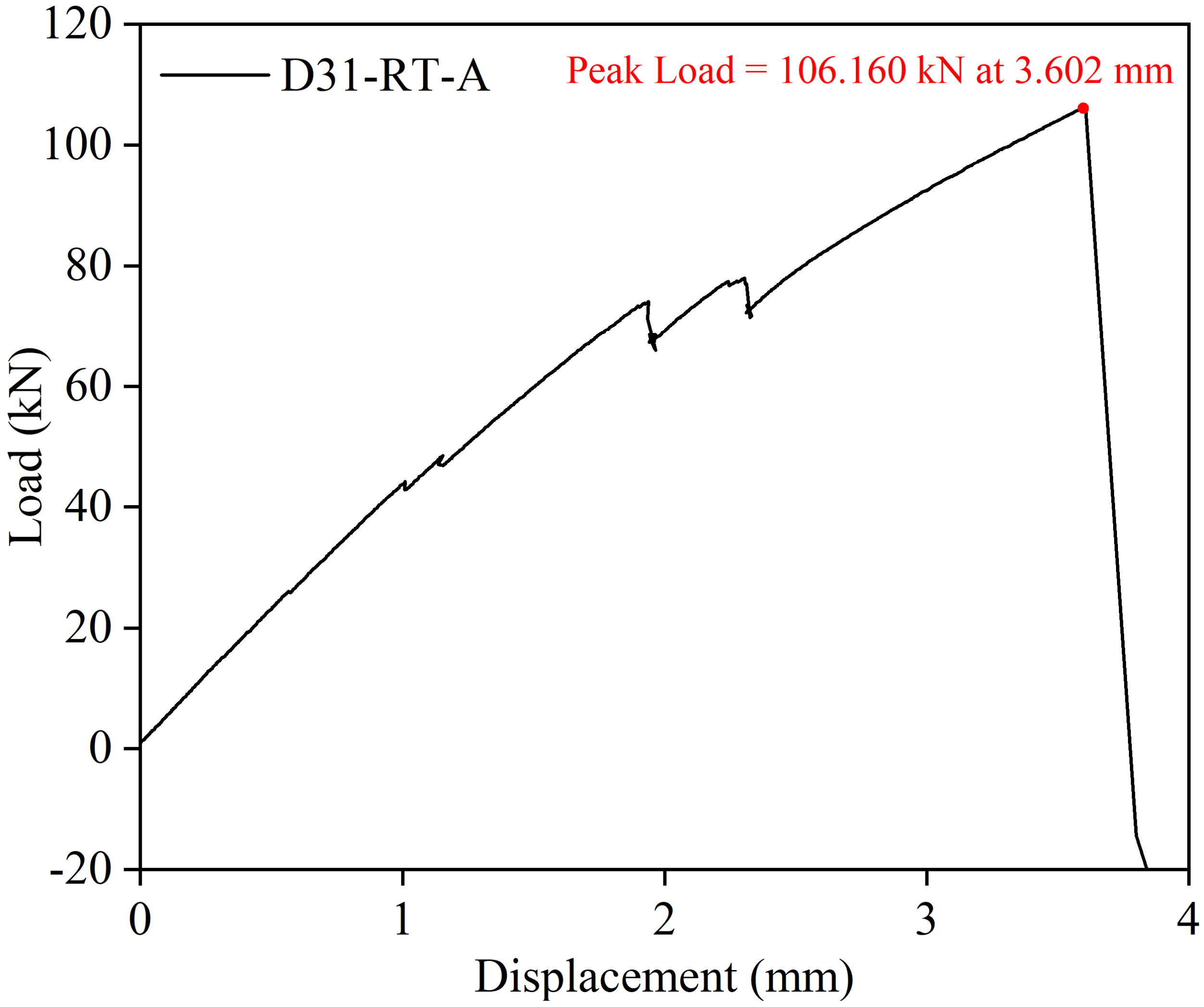

Figure S5. Complete D31-RT-A load–machine-displacement record. The maximum is 106.160 kN at 3.602 mm. Pronounced intermediate load drops are retained as recorded; without synchronized local imaging, they are not assigned to a particular interface or tube end. The negative post-failure portion of the untrimmed machine trace is not used in the peak analysis.
